# Physiological and Proteomic Signatures Reveal Mechanisms of Superior Drought Resilience in Pearl Millet Compared to Wheat

**DOI:** 10.3389/fpls.2020.600278

**Published:** 2021-01-13

**Authors:** Arindam Ghatak, Palak Chaturvedi, Gert Bachmann, Luis Valledor, Živa Ramšak, Mitra Mohammadi Bazargani, Prasad Bajaj, Sridharan Jegadeesan, Weimin Li, Xiaoliang Sun, Kristina Gruden, Rajeev K. Varshney, Wolfram Weckwerth

**Affiliations:** ^1^Molecular Systems Biology Lab (MOSYS), Department of Functional and Evolutionary Ecology, University of Vienna, Vienna, Austria; ^2^Plant Physiology Lab, Organisms and Systems Biology, Faculty of Biology, University of Oviedo, Oviedo, Spain; ^3^Department of Systems Biology and Biotechnology, National Institute of Biology, Ljubljana, Slovenia; ^4^Agriculture Institute, Iranian Research Organization for Science and Technology, Tehran, Iran; ^5^Center of Excellence in Genomics & Systems Biology, International Crops Research Institute for the Semi-Arid Tropics, Hyderabad, India; ^6^Premas Life Sciences, Bengaluru, India; ^7^Vienna Metabolomics Center (VIME), University of Vienna, Vienna, Austria

**Keywords:** climate resilience, senescence, cereals, drought stress, proteomics, stay-green trait, secure food production, marker assisted breeding

## Abstract

Presently, pearl millet and wheat are belonging to highly important cereal crops. Pearl millet, however, is an under-utilized crop, despite its superior resilience to drought and heat stress in contrast to wheat. To investigate this in more detail, we performed comparative physiological screening and large scale proteomics of drought stress responses in drought-tolerant and susceptible genotypes of pearl millet and wheat. These chosen genotypes are widely used in breeding and farming practices. The physiological responses demonstrated large differences in the regulation of root morphology and photosynthetic machinery, revealing a stay-green phenotype in pearl millet. Subsequent tissue-specific proteome analysis of leaves, roots and seeds led to the identification of 12,558 proteins in pearl millet and wheat under well-watered and stress conditions. To allow for this comparative proteome analysis and to provide a platform for future functional proteomics studies we performed a systematic phylogenetic analysis of all orthologues in pearl millet, wheat, foxtail millet, sorghum, barley, brachypodium, rice, maize, Arabidopsis, and soybean. In summary, we define (i) a stay-green proteome signature in the drought-tolerant pearl millet phenotype and (ii) differential senescence proteome signatures in contrasting wheat phenotypes not capable of coping with similar drought stress. These different responses have a significant effect on yield and grain filling processes reflected by the harvest index. Proteome signatures related to root morphology and seed yield demonstrated the unexpected intra- and interspecies-specific biochemical plasticity for stress adaptation for both pearl millet and wheat genotypes. These quantitative reference data provide tissue- and phenotype-specific marker proteins of stress defense mechanisms which are not predictable from the genome sequence itself and have potential value for marker-assisted breeding beyond genome assisted breeding.

## Introduction

Feeding nine billion people with balanced nutritional diet under unpredictable severe weather events is a challenging task. Emerging evidence suggests that the climate change crisis will cause shifts in food production and yield loss, causing a severe threat to food security (Lunt et al., 2016). A key strategy to adapt in a changing climate is to develop elite germplasms that can survive under hostile weather conditions with stable yields and to promote underutilized crop species. Focusing and exploiting the large reservoir of minor and underutilized crop plants would provide a more diversified agricultural system and an alternative healthy food resource, ensuring food, and nutritional security (Mabhaudhi et al., 2019).

The world today relies on a small number of crop species for food, majorly C_3_ cereals (wheat, rice, barley), and few C_4_ cereals like pearl millet, maize and sorghum (Alexandratos and Bruinsma, 2012). By far, the abundance of genetic resources and potentially beneficial traits of C_4_ cereals are neglected. However, to alleviate the food crisis, efforts are ongoing to engineer C_4_ traits into C_3_ crop species, which can massively increase C_3_ crop yields (von Caemmerer et al., 2012; Weissmann and Brutnell, 2012; Wang et al., 2014; Rangan et al., 2016). However, such efforts require an improved understanding of the physiological traits (such as deep rooting, stay-green, and senescence etc.) and system-level analysis to identify the regulatory networks underlying these physiological traits under abiotic stress condition in a comparative manner.

Drought can adversely and drastically affect the agricultural sector. It causes land degradation and biodiversity loss. Every year, around 8.5 million ha of rain-fed land and 1.5 million ha of irrigated lands are affected because of salinization (Hanjra and Qureshi, 2010). Drought induces profound changes at the morphological, physiological and biochemical level in all plant tissues (Anjum et al., 2011), mostly disturbing the complicated relationship between sink and source of plant organs. Upon perception of drought stress a complex response is initiated which includes massive transcriptional reprogramming along with anatomical and physiological alterations which include deep root system, changes in leaf morphology, closure of stomata, cuticular wax thickening, hormone induction, reactive oxygen species (ROS) scavenging, osmolyte synthesis, nitrogen assimilation, and amino acid metabolism (Lamalakshmi Devi et al., 2017). These active processes involve genes, proteins and small molecules (metabolites), which play a crucial role in shaping the final phenotype of the plants (Ghatak et al., 2018; Weckwerth et al., 2020). However, response to drought is species-specific and often genotype-specific (Campos et al., 2004). It also largely depends upon the duration and severity of water loss, age, and stage of the plant development (Pinheiro and Chaves, 2011).

Proteomics has become a powerful tool for analyzing plant response to various environmental stimuli (biotic and abiotic), especially in the comparative studies of genetically diverse germplasms subjected to drought stress, providing fundamental insights into plant responses to pre-determined stress and biochemical pathways that participate in the acclimatization process. Proper evaluation of the data can contribute to the identification of the potential candidates, which are then correlated with the quantitative trait loci (QTLs). These candidates can be further integrated into the marker-assisted breeding strategy to enhance the selection of plants with desired traits (Tuberosa and Salvi, 2006). Several studies were performed to understand the effect of drought stress on crop plants at proteomics level (Riccardi et al., 2004; Ford et al., 2011; Komatsu et al., 2014; Chmielewska et al., 2016; Ghatak et al., 2016; Ghatak et al., 2017a, b; Michaletti et al., 2018; Rodziewicz et al., 2019).

Wheat (*Triticum aestivum* L.) and pearl millet (*Pennisetum glaucum* (L.) R. Br.) are most important cereal crops. Wheat is a C_3_ cereal crop with a hexaploid genome (∼17 Gb) (Appels et al., 2018). It is a food source of > 50% world population. The yield of wheat is severely compromised under harsh climatic condition, especially drought (Ahmed et al., 2020). Contrastingly, pearl millet is a C_4_ grass highly cross-pollinated diploid (2n = 2x = 14) with a relative genome of 1.79 Gb and high photosynthetic efficiency (Varshney et al., 2017). It is an underutilized crop, despite its immense nutritional potential which has not been tapped. Unlike wheat, pearl millet is cultivatable in areas with drought, low soil fertility, high salinity, low pH or high temperature. As compared to other cereals, pearl millet showed greater ceiling temperatures for grain yield, making it a climate-resilient crop suitable for semi-arid regions of the world (Varshney et al., 2017). Projected changes in crop yields owing to climate change demand a paradigm shift to enhance the cultivation and distribution of such crops in the market reducing the burden of the crops with high commercial value, e.g., wheat and maize without compromising their nutritional importance. However, there is a lack of studies that provide insights into the molecular machinery underlying stress tolerance in millets in comparison to other important cereals.

To address this aim, in the present study comparative physiological and proteome changes were evaluated in the leaves, roots and seeds of two different pearl millet and wheat genotypes from different geographical origin subjected to drought: (1) to identify physiological traits associated with tolerance to the deleterious effect of drought stress, (2) to characterize physiological traits such as photosynthetic activity, root length, seed weight and weight of the plant with contrasting degree of drought tolerance, (3) to explore the implications of drought stress on proteome, and identify tissue-specific (roots, leaves and seeds) drought stress-responsive proteins which attribute to the stress tolerance of these crops, (4) to identify and compare abundance profile of the proteins involved in C_4_ pearl millet photosynthetic metabolism and wax biosynthesis with the orthologous proteins present in C_3_ wheat, and (5) to integrate physiological and biochemical parameters (i.e., identified proteins) using multivariate analysis to obtain a comprehensive picture of the plants “physiological trait/proteome levels” under drought stress.

## Materials and Methods

### Plant Material, Growth Conditions, and Drought Treatment

Two different genotypes of pearl millet (843-22B and ICTP8203) and spring wheat (White Fife and TRI 5630) from different geographic origins (Table 1A) were selected for this study: wheat genotypes, one originating from the United Kingdom, accession number TRI 5357 (White Fife, here indicated as UK), the other from Iran, accession number TRI 5630 (indicated as IR). Pearl millet genotypes originated from different states of India primarily used for breeding and research. Seeds were obtained from the gene bank repository of International Crops Research Institute for the Semi-Arid Tropics (ICRISAT), India and IPK, Germany. The experiment started in February and concluded in July. Plants were grown in a controlled condition: the temperature was max 30°C during the daytime, 26°C at nighttime (±2°C). Relative air humidity was 60% during the day, and 80% at nighttime; the light was provided by metal halide lamps (HRI-TS 250W/NDL Neutral white, Radium, Germany) at an intensity of 220 μmol photons m^–^^2^ s^–^^1^ (7 a.m. to 9 p.m). The plants were grown in custom made cylindrical pipes (Ghatak et al., 2016). Each pipe was made of 5 polyethylene segments (15 cm each) amounting to a total height of 75 cm with an inner diameter of 10.3 cm. The total soil volume was 6.25 L. Each tube had two access openings (one in the upmost segment, and one in the 2nd segment from the bottom) for monitoring soil dehydration (monitored by measuring the volumetric soil water content (volume of water/total volume ratio), in percentage) and soil temperature by means of 1% accurate theta probes by (ADC ML3^TM^) sensors. Soil mixture consisted of three parts of potting ground (peat, humus), 2 parts of sand, 1 part of styromull (Royal Brinkman, the Netherlands) and 0.1 % NPK was added as initial fertilizer and no pesticides were used.

**TABLE 1A:** Description of the genotypes used in the study.

Wheat *Triticum aestivum* L.		Pearl millet *Pennisetum glaucum* (L.) R. Br.	
United Kingdom (UK)	IRAN (IR)	Telangana, India	Maharashtra, India
White Fife	TRI 5630	843-22B	ICTP8203
Sensitive	Tolerant	Sensitive	Tolerant

**FIGURE 1:**
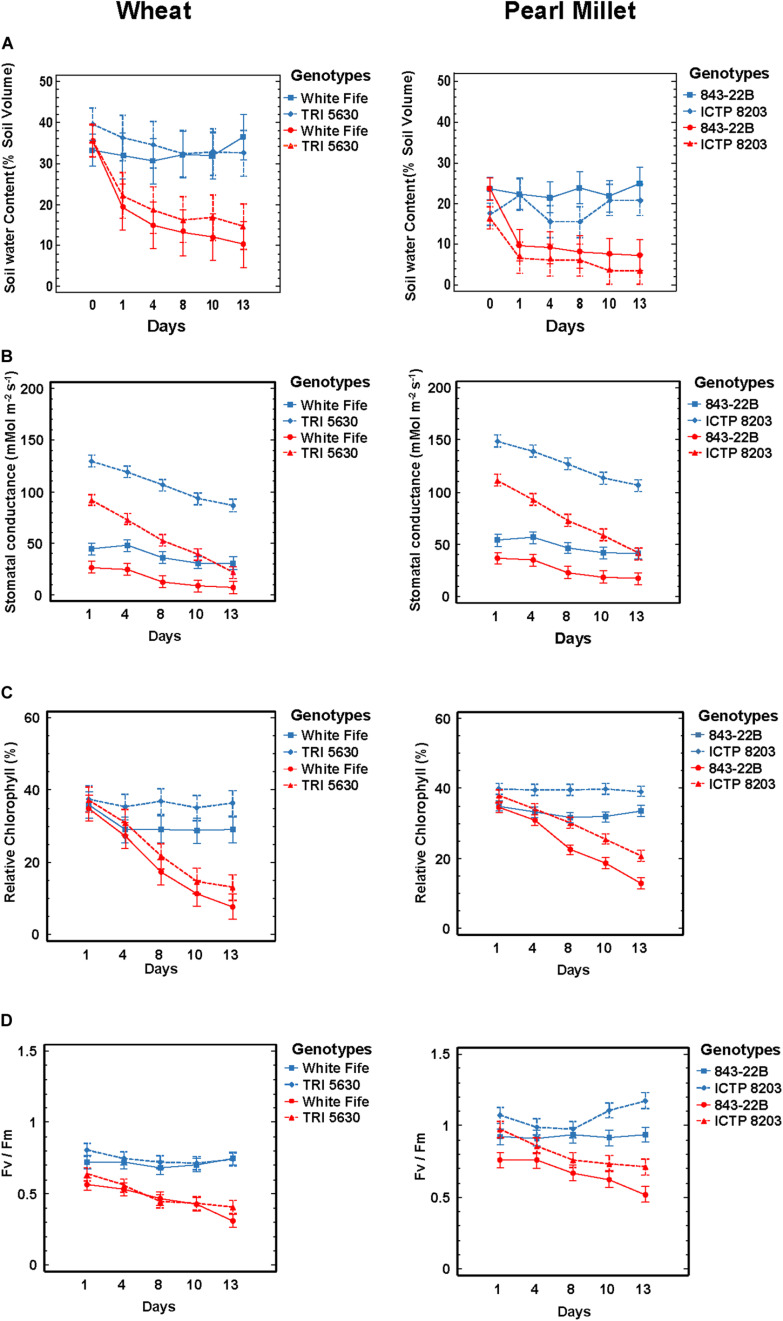
Physiological parameters. **(A)** Soil moisture content was measured using sensors ML 3 ThetaProbe. **(B)** Stomatal conductance was measured using PWMR-4 Porometer. **(C)** Leaf chlorophyll content was determined using SPAD chlorophyll meter. **(D)** Chlorophyll fluorescence (F_v_/F_m_) was determined using plant efficiency analyzer (PEA). All the analysis was performed under control and stress condition in pearl millet and wheat genotypes (color indication: blue—control and red—drought stress; lines: dotted lines—tolerant genotypes and direct line—sensitive genotypes).

The irrigation was adapted to the plant physiological needs, i.e., shoot/root development and evapotranspiration, being higher for wheat, and lower for pearl millet (Figure 1A). As a consequence, the wheat plants were kept at ∼32% of soil volume (71.11% of field capacity), and pearl millet plants at ∼24% of soil volume (53.33% of field capacity). The drought stress began when the plants reached the developmental stage of phase 51–53 on the BBCH scale, which was achieved in 8 weeks for pearl millet and 10 weeks for wheat. The difference in soil water content between control and stressed plants was the first indication of the drought imposed. The plant material (roots, leaves and seeds) were collected considering four biological replicates in each condition (control and stress) for proteomic analysis after 13-days of drought period. The harvested samples were frozen in liquid nitrogen to stop any enzymatic activity. The tissue samples were ground in liquid nitrogen using mortar and pestle. Pulverized tissues were stored at −80°C until further analysis. Table 1B provides the details of the genotypes, harvested tissues and their abbreviations used in the manuscript text, figures and tables.

**TABLE 1B:** Details of the genotypes, tissues harvested and abbreviations used in the figures and tables.

Cereal crops	Genotype name	Genotype	Conditions applied	Tissues harvested	Abbreviations for figures and tables
Wheat	White Fife	Sensitive	Control (C)	Root (R)	UK-C-R
	(indicated as UK)			Seed (S)	UK-C-S
				Leaf (L)	UK-C-L
			Stress (St)	Root (R)	UK-St-R
				Seed (S)	UK-St-S
				Leaf (L)	UK-St-L
	TRI 5630	Tolerant	Control (C)	Root (R)	IR-C-R
	(indicated as IR)			Seed (S)	IR-C-S
				Leaf (L)	IR-C-L
			Stress (St)	Root (R)	IR-St-R
				Seed (S)	IR-St-S
				Leaf (L)	IR-St-L
Pearl	843-22B	Sensitive	Control (C)	Root (R)	PM-S-C-R
Millet (PM)		(S)		Seed (S)	PM-S-C-S
				Leaf (L)	PM-S-C-L
			Stress (St)	Root (R)	PM-S-St-R
				Seed (S)	PM-S-St-S
				Leaf (L)	PM-S-St-L
	ICTP8203	Tolerant	Control (C)	Root (R)	PM-T-C-R
		(T)		Seed (S)	PM-T-C-S
				Leaf (L)	PM-T-C-L
			Stress (St)	Root (R)	PM-T-St-R
				Seed (S)	PM-T-St-S
				Leaf (L)	PM-T-St-L

### Physiological Measurements

#### Stomatal Conductance, Leaf Chlorophyll Content, and Chlorophyll Fluorescence

The effect of drought stress was examined by measuring stomatal conductance (g_s_) (mmol m^–^^2^ s^–^^1^) using PWMR-4 porometer (PP Systems, United States), leaf chlorophyll content using SPAD chlorophyll meter (SPAD 502, Minolta, Tokyo), and chlorophyll fluorescence (F_v_/F_m_) using plant efficiency analyzer (PEA) (Handy PEA, Hansatech Instruments, King’s Lynn, United Kingdom). Mature and fully expanded green leaves were used for the measurement at regular interval until the drought treatment was completed. The measurements were performed non-destructively on plant attached leaves.

#### Plant Weight (Biomass), Root Length, Panicle/Spike Characteristic (Numbers of Panicle/Spike per Biological Replicate), Seed Weight, and Harvest Index

For the measurement of plant weight (biomass), the cylindrical pipes were dismantled, and the intact plant was removed carefully from the soil. Panicles and spikelets on culms were counted; seed weight was recorded before putting them into liquid nitrogen. Harvest index (HI) was calculated according to Schauer et al. (2006) with the formula:

Harvest index (HI) % = [Total yield (i.e., seed weight)/Total yield + plant weight (i.e., biomass)] × 100

#### Protein Extraction and Pre-fractionation

The total protein from roots, leaves and seeds was extracted according to Chaturvedi et al. (2013) and Valledor and Weckwerth (2014). In brief, homogenized tissue weighed (20 mg for root, leaf and seed tissue, respectively), and suspended in 200 μL of protein extraction buffer [100 mM Tris- HCl, pH 8.0; 5% SDS, 10% glycerol; 10 mM DTT; 1% plant protease inhibitor cocktail (Sigma P9599)] and incubated at room temperature for 5 min followed by incubation for 2.5 min at 95°C and centrifugation at 21,000 × g for 5 min at room temperature. The supernatant was carefully transferred to a new tube. Two-hundred microliters of 1.4 M sucrose were added to the supernatant and proteins were extracted twice with 200 μL TE buffer-equilibrated phenol followed by counter extraction with 400 μL of 0.7 M sucrose. Phenol phases were combined and subsequently mixed with 2.5 volumes of 0.1 M ammonium acetate in methanol for precipitation of proteins. After 16 h of incubation at −20°C, samples were centrifuged for 5 min at 5,000 × g. The pellet was washed twice with 0.1 M ammonium acetate, once with acetone and air-dried at room temperature. The pellet was re-dissolved in 6 M Urea and 5% SDS, and protein concentration were determined using the bicinchoninic acid assay (BCA method). Proteins were pre-fractionated by SDS-PAGE. Forty micrograms of total protein were loaded onto the gel. Gels were fixed and stained with methanol: acetic acid: water: Coomassie Brilliant Blue R-250 (40:10:50:0.001). Gels were destained in methanol: water (40:60).

#### Protein Digestion and LC−MS/MS

Gel pieces were destained, equilibrated and digested with trypsin, desalted and concentrated (Chaturvedi et al., 2013). Prior to mass spectrometric measurement, the tryptic peptide pellets were dissolved in 4% (v/v) acetonitrile, 0.1% (v/v) formic acid. One μg of the digested peptide from each tissue sample (4 biological replicates for each condition) was loaded on a C18 reverse-phase column (Thermo scientific, EASY-Spray 500 mm, 2 μm particle size). Separation was achieved with a 90 min gradient from 98% solution A (0.1% formic acid) and 2% solution B (90% ACN and 0.1% formic acid) at 0 min to 40% solution B (90% ACN and 0.1% formic acid) at 90 min with a flow rate of 300 nL min^–^^1^. nESI-MS/MS measurements were performed on Orbitrap Elite (Thermo Fisher Scientific, Bremen, Germany) with the following settings: Full scan range 350–1,800 m/z resolution 120,000 max. 20 MS2 scans (activation type CID), repeat count 1, repeat duration 30 s, exclusion list size 500, exclusion duration 30 s, charge state screening enabled with the rejection of unassigned and +1 charge states, minimum signal threshold 500.

#### Peptide and Protein Identification

Raw data were searched with the SEQUEST algorithm present in Proteome Discoverer version 1.3 (Thermo, Germany) as described in Valledor and Weckwerth (2014). We have used the following settings in Proteome Discoverer for data analysis which include: Peptide confidence: High, which is equivalent to 1% false discovery rate (FDR), and Xcorr of 2, 3, 4, 5, 6 for peptides of charge 2, 3, 4, 5, 6. The variable modifications were set to acetylation of N-terminus and oxidation of methionine, with a mass tolerance of 10 ppm for parent ion and 0.8 Da for the fragment ion. The number of missed and non-specific cleavages permitted was 2. There were no fixed modifications, as dynamic modifications were used.

For identification, newly annotated pearl millet genome database containing 38,579 genes (Varshney et al., 2017) and UniProt database containing the annotations of 136,865 genes for wheat was used. Peptides were matched against these databases plus decoys, considering a significant hit when the peptide confidence was high. All the MS/MS spectra of the identified proteins and their meta-information were further uploaded to PRIDE repository. Sample codes for the raw files deposited in the PRIDE are provided in Supplementary Table S1. Submission details are as follows; Project name: Comparative physiological and proteomic signatures reveal contrasting stay-green and senescence phenotypes in drought tolerant and susceptible pearl millet and wheat genotypes. Project accession: PXD021446.

The identified proteins were quantitated based on total ion count, followed by an NSAF normalization strategy (Paoletti et al., 2006):

$${\left(NSAF\right)}_{k}={\left(PSM/L\right)}_{k}/\Sigma_{i=1}^{N}{\left(PSM/L\right)}_{i}$$

In which the total number spectra counts for the matching peptides from protein k (PSM) was divided by the protein length (L), then divided by the sum of PSM/L for all N proteins.

#### Statistical Analysis and Data Integration

Statistical analysis for physiological data points was performed using Statgraphics (ver. 17.2.05) and SIMCA (version 13) for OPLS-DA analysis. For both PCA and OPLS-DA, data were centered and scaled using z-transformation.

#### Bioinformatics for Functional Annotation

To assign functional descriptions to pearl millet and wheat sequences, BLAST search (Altschul et al., 1997) was performed against Arabidopsis proteins release Araport11 (Cheng et al., 2017), rice (Kawahara et al., 2013), tomato SL3.0_ITAG3.2 (Sato et al., 2012), potato (Xu et al., 2011), and plants in Swiss-Prot (Bateman et al., 2017) using default settings. Every accession was assigned one best match (alignment coverage of shorter sequence ≥ 70%; E-value ≤ 10^−10^), prioritizing Arabidopsis and rice matches over the rest, when available. Assignment of pearl millet and wheat accession with MapMan plant functional ontology terms (Thimm et al., 2004) was also based on BLAST results against the same databases (alignment coverage of shorter sequence ≥ 70%, E-value ≤ 10^−20^, bit score ≥ 50, positives % ≥ 60). Pearl millet and wheat accessions then inherited the BIN assignment from the corresponding best match; unmatched sequenced were assigned BIN 35.2 (not assigned. unknown). To enable visualization of high-throughput experimental results, MapMan and GSEA mapping files were created for each species (available from www.gomapman.org/export/current/, Ramsak et al., 2014).

#### Gene Family and Phylogenetic Analysis

For gene family analysis of 11 plant species, DIAMOND (Buchfink et al., 2015) was used with an *e*-value cutoff of ≤ 1.0e-05. In addition to pearl millet, wheat sequences were downloaded from Swiss-Prot, while PLAZA v4 resource (Van Bel et al., 2018) was used for *Arabidopsis thaliana*, *Brachypodium distachyon*, *Glycine max*, *Hordeum vulgare*, *Oryza sativa* ssp. *japonica*, *Oryza sativa* ssp. *indica*, *Sorghum bicolor*, *Setaria italic*, and *Zea mays*. To reduce redundancy present in wheat sequences, these were pre-processed using CD-HIT (Fu et al., 2012) (≥80% identity; ≥80% coverage for shorter and longer sequence compared). The proteins were clustered using OrthoMCL v2.0.9 (Li et al., 2003), to define gene families with paralogs and orthologs. Single copy genes in an OrthoMCL cluster for all species were used to construct a phylogenetic tree in SeaView with muscle for multiple sequence alignment, Gblock to select the conserved regions and PhyML to construct the phylogenetic tree (bootstrap 1000).

#### Statistics for Proteome Data Analysis

Data were normalized using normalized spectral abundance factor (NSAF) approach and subjected to multivariate (Principal components analysis (PCA), K-means clustering) analysis which was performed using the statistical toolbox COVAIN in MATLAB (Sun and Weckwerth, 2012) and univariate (two-way ANOVA) analysis was performed considering two factors, treatment (control and stress), genotypes (pearl millet and wheat) and their interactions. Each table consists of df (degree of freedom), *F*-value (*F*-test) and *p*-value (of the *F*-test) for every factor. For K-means clustering analysis, proteins were chosen only if they were present in all four biological replicates of at least one condition. All the identified proteins were categorized into functional groups to allow a functional view of the tissue-specific proteome. The sum of the NSAF values for each functional category was then visualized using spider plots. Sparse partial least squares (sPLS), discriminant and network analysis were performed to integrate physiological parameters and proteome data to show the interaction between proteins (predictors) and physiology (response). sPLS was performed employing R package mixOmics. Generated networks were visualized and filtered (only edges equal or higher than 0.9 were maintained) in Cytoscape v.2.8.3 (Escandon et al., 2017).

The Venn diagrams were produced using GeneVenn^1^. A protein was considered as differentially expressed between two samples if three conditions were met: (1) the protein was detected in all four replicates at least in one of the treatments, (2) *p*-value for differential expression was ≤ 0.01 and (3) the fold change in protein NSAF values between the samples was at least 1.5. Volcano and spider plots were produced using Microsoft Excel 2015. Box plots were constructed using program R (version 3.5, R Core Team 2019) (package ggplot2).

## Results

### Genotypic Variation of Physiological Responses, Plant Biomass, and Yield Under Drought Stress

In order to investigate the physiological basis of genotypic variation under drought stress, several parameters were determined, including stomatal conductance, F_v_/F_m_, root growth and others (see section “Materials and Methods”). All the recorded observations are provided in Supplementary Table S2.

Principal component analysis (PCA) and orthogonal partial least squares discriminant analysis (OPLS–DA) was performed considering all the factors and variables of the physiological data (Supplementary Figure S1A). A clear separation was observed between pearl millet and wheat genotypes on discriminant function 2 (PC2) as well as between control and stress condition of each genotype on discriminant function 1 (PC1). Several physiological parameters separated individual genotypes. The data revealed that the growth parameter of all the compared genotypes was affected differently. The PLS-DA emphasizes the differential diagnostic values discriminating between the genotypes and treatments. Stomatal conductance and photosynthetic parameters such as F_v_/F_m_ and chlorophyll measurements (SPAD) discriminated most between control and stressed plants, whereas the number of the panicles and spikelets was the highest discriminant value between the genera *Pennisetum* and *Triticum* as observed in the PCA (Supplementary Figure S1B).

The determination of basic growth parameters is essential in the characterization of drought stress response mechanisms (Jones, 2007). Overall, plant biomass was decreased under stress condition in all the four genotypes compared to controls (Supplementary Table S2). The highest reduction in biomass was observed in TRI 5630, followed by ICTP8203 (Supplementary Table S2), but they also showed the highest seed yield under drought. Stressed plants of the wheat genotypes have shown decrease in the seed weight compared to the pearl millet genotypes and the most severe effect was observed in White Fife which is the most susceptible genotype to drought stress (Supplementary Table S2).

### Contrasting Genotype Effects of Stomata Responses to Drought Stress

The immediate response of plants under drought stress is stomatal closure to prevent water loss via transpiration (Buckley, 2019). Plants grown under drought conditions tend to have lower stomatal conductance, thus helping to conserve water and maintain an adequate leaf water status but at the same time reducing leaf internal CO_2_ concentration and photosynthesis. The precise relationship is also dependent on other factors, like genotypes, drought history and environmental conditions. We measured stomatal conductance (gs) at regular intervals from the start until the conclusion of the stress treatment, considering fully grown leaves using a PWMR-4 porometer. It was observed that control plants of tolerant genotypes TRI 5630 and ICTP8203 showed stomatal conductance ranging between 130 and 150 mmol m^–^^2^s^–^^1^ and sensitive genotypes (White Fife and 843-22B) showed conductance between 40 and 50 mmol m^–^^2^s^–^^1^ (Figure 1B and Supplementary Table S2). Stomatal conductance of the stressed plants declined as drought stress progressed. In the sensitive genotypes White Fife and 843-22B, the conductance declined gradually and reached 10 mmol m^–^^2^s^–^^1^. Interestingly, the most significant and rapid effect was observed in the tolerant genotypes TRI 5630 and ICTP8203 where stomatal conductance reached 20 and 40 mmol m^–^^2^s^−1^, respectively, at the end of drought treatment (Figure 1B).

### Contrasting Regulation of Chlorophyll Content and F_v_/F_m_ Under Drought Stress Revealed a Stay-Green Phenotype vs. Senescence Phenotypes in Pearl Millet and Wheat

In order to understand the photosynthetic capabilities of pearl millet and wheat genotypes, leaf chlorophyll content was determined using a SPAD meter (Figure 1C and Supplementary Table S2). The SPAD values in the stressed plants declined as the drought stress progressed. In the control condition, SPAD value ranged between 20 and 40% approximately for both the genotypes of pearl millet and wheat. Under dehydration state, SPAD value reduced to 10–24% approximately. The highest chlorophyll content recorded during stress was in ICTP8203 (24%), followed by 843-22B (14%), TRI 5630 (14%), and White Fife (>10%).

Drought stress consistently and significantly reduced the maximum efficiency of PSII photochemistry (F_v_/F_m_), though this effect varied in its severity among the different genotypes (Figure 1D and Supplementary Table S2). By the end of the drought regime, the highest F_v_/F_m_ ratio was observed in ICTP8203, followed by 843-22B and TRI 5630. This correlates directly with the SPAD measurements, indicating the stay-green phenotype of ICTP8203 in contrast to the other genotypes. The lowest reading was recorded in White Fife (Figure 1D).

### Comparative Analysis of Tissue-Specific Pearl Millet and Wheat Drought Stress Proteomes

**FIGURE 2:**
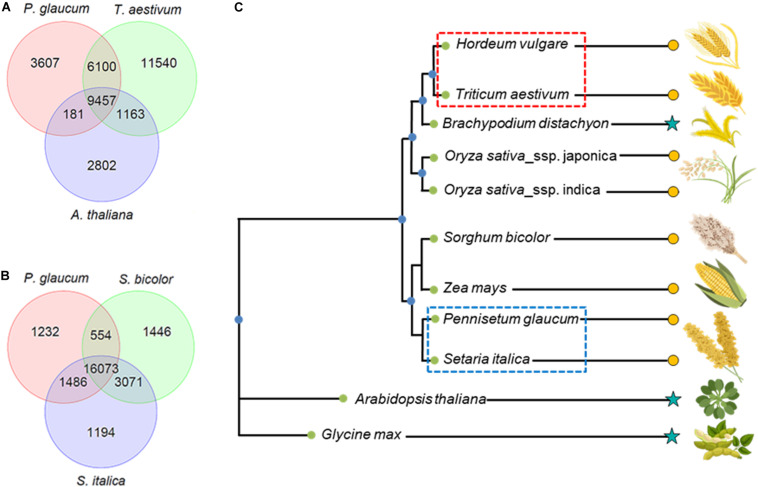
Comparative analysis of pearl and wheat proteome with other species and phylogenetic tree resembling their nearest ortholog via OrthoMCl software search. **(A)** Venn diagram: Shared genes between pearl millet, wheat and Arabidopsis. **(B)** Venn diagram: Shared genes between pearl millet, sorghum, and foxtail millet. **(C)** Phylogenetic tree comparing relative orthologue in barley, wheat, brachypodium, rice, sorghum, maize, foxtail millet, pearl millet, arabidopsis, and soybean (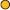, cereal crops; 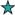, other plant species).

To perform a detailed proteome study, not only a full genome sequence is required, but also accurate gene annotation plays a critical role (Valledor et al., 2012). Here, for a comparative proteomics study, we identified unique and shared gene families between pearl millet and wheat using OrthoMCL (Li et al., 2003). Because of the high redundancy of the wheat genome annotation, the wheat sequences were clustered using CD-HIT (Fu et al., 2012), resulting in a set of 69,215 sequences (from the initial 136,866). From the total 46,954 gene families detected by the OrthoMCL algorithm, 9,457 were found to be shared between pearl millet, wheat and *Arabidopsis* (Figure 2A). On the gene level, ∼40% of the proteomes was shared between *Arabidopsis* and pearl millet (13,710 proteins for *Arabidopsis* and 11,535 proteins for pearl millet). Comparative analysis was also performed considering sorghum *(Sorghum bicolor)* and foxtail millet (*Setaria italica)* (Figure 2B). In the comparison between pearl millet and *sorghum*, 70–75% of the proteome was shared (19,865 proteins for pearl millet and 20,279 proteins for sorghum). Foxtail millet represents the evolutionarily closest plant species to pearl millet in this ortholog family analysis (Figure 2C, blue). Between these two species, ∼70–80% of the proteome was shared (21,447 proteins for pearl millet and 22,170 proteins for foxtail millet). For wheat, the closest related plant species is barley (*Hordeum vulgare)* (Figure 2C, red), where coverage of the former is 59% (30,624 proteins of the reduced redundancy sequence set) and 94% for the latter (18,239 proteins).

From all the detected peptides in roots, leaves and seeds, 12,558 proteins were identified from both pearl millet and wheat genotypes, of which 4,564 proteins were identified in pearl millet (843-22B and ICTP8203) genotypes (Supplementary Table S3). In wheat genotypes (White Fife and TRI 5630), 7,994 proteins were identified (Supplementary Table S4). In order to generate a broad survey of identified proteins with altered tissue-specific abundance under drought stress, a Venn analysis was conducted which determines the dynamics of the proteome in selected genotypes of pearl millet and wheat under control and drought stress (Supplementary Figure S2). We performed two-way ANOVA analysis of the identified pearl millet and wheat proteome for every factor: (i) treatment (control and stress) and, (ii) genotypes (pearl millet and wheat) and (iii) their interactions. Here, each tissue (root, leaf and seed) was analyzed separately (Supplementary Table S5).

### Functional Categorization and Statistical Analysis of the Drought Stress Proteome in Contrasting Pearl Millet and Wheat Genotypes

**FIGURE 3:**
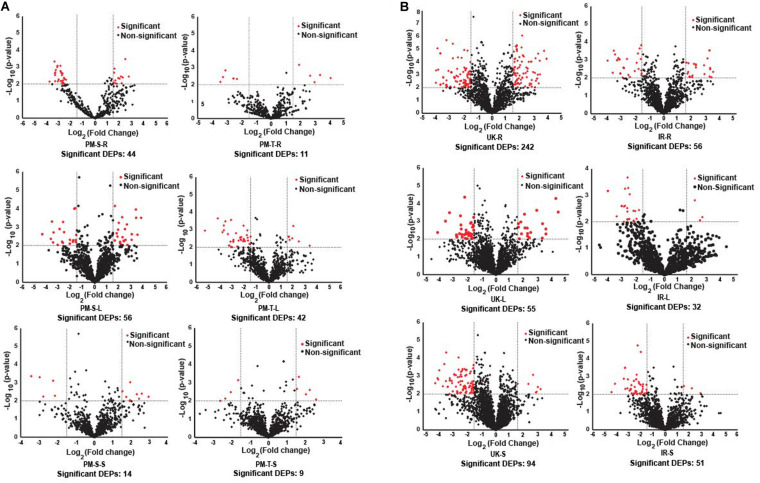
Volcano plots. **(A)** Represents differentially expressed proteins (DEPs) in root, leaf and seed tissues of pearl millet genotypes. **(B)** Represents differentially expressed proteins (DEPs) in root, leaf and seed tissues of wheat genotypes.

The tissue-specific DEPs (differentially expressed proteins) in pearl millet (843-22B and ICTP8203) and wheat (White Fife and TRI 5630) genotypes are represented using volcano plot’s (Figures 3A,B), and the list of DEPs are summarized in Supplementary Tables S6, S7. K-means clustering analysis was employed to investigate the co-expression/abundance pattern of the identified proteins from the compared genotypes. For cluster analysis, proteins were considered if they were present in all the four biological replicates of at least in one tissue/condition. Tissue-specific grouping of proteins in different condition (control and stress) lead us with clusters *k* = 30 in 843-22B and *k* = 35 in ICTP8203 (Supplementary Table S8), similarly, *k* = 50 in White Fife and TRI 5630 (Supplementary Table S9). Cluster analysis revealed specific groups of proteins with changing abundance in tissue or drought stress condition.

**FIGURE 4:**
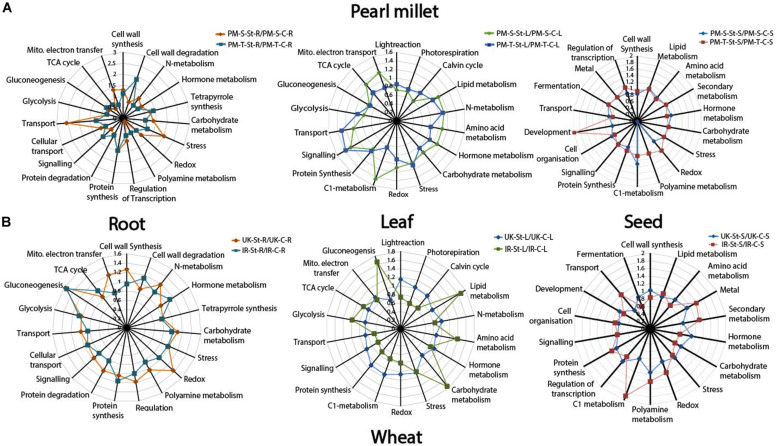
Spider plots. **(A)** Represents functional distribution of the proteome in the root, leaf, and seed tissues of pearl millet genotypes. **(B)** Represents functional distribution of the proteome in the root, leaf, and seed tissues of wheat genotypes.

Functional categorization of the identified proteins was performed according to Ramsak et al. (2014). Tissue-specific functional distribution of the proteome for pearl millet (843-22B and ICTP8203) and wheat (White Fife and TRI 5630) genotypes under drought stress is depicted in Figure 4 via spider plots using the total NSAF score summed up for different functional categories (Chaturvedi et al., 2013, 2015). The overall pattern demonstrates a remarkable variation of proteome functionality between the sensitive and tolerant-genotypes expressed as ratios of drought stress vs. controls. In pearl millet, major enhanced functional categories in 843-22B are transport and stress-related proteins in the root, mitochondrial electron transport, TCA cycle, C1-metabolism in leaf. In contrast, ICTP8203 showed drought stress enhanced protein functions for cell wall degradation, signaling and polyamine metabolism in the root, light reactions, photorespiration, transport and signaling in leaf and a strong regulation was observed in the development and polyamine metabolism in seed tissue. In wheat, White Fife showed enhanced regulation in protein categories of cell wall synthesis, mitochondrial electron transport, and redox in root tissue. In the leaf, TRI 5630 showed pronounced proteome regulation in the functional categories of gluconeogenesis, lipid metabolism, amino acid metabolism and carbohydrate metabolism compared to the White Fife. Similarly, seed proteome of TRI5630 showed enhanced proteome regulation compared to White Fife, e.g., C1 metabolism, secondary metabolism and transport (Figure 4). Functional categories and related proteins distinguishing the genotype- and tissue-specific drought stress response according to Figure 4 can be found in Supplementary Tables S10, S11.

Principal component analysis (PCA) of protein NSAF scores was performed using COVAIN (Sun and Weckwerth, 2012). All the tissues were separated by the first principal component (PC1) providing hints for tissue-specific proteome functionality in both pearl millet and wheat. In pearl millet, the strongest variation was observed in root and leaf tissues (Supplementary Figure S3A). In PCA of ICTP8203, leaf proteome showed the strongest variation in response to drought stress compared to roots and seeds (Supplementary Figures S3B,C). Positive loadings of PC1 represent proteins with higher abundances in roots, whereas negative loadings depicted higher levels in leaf and seed tissues (Supplementary Table S12). In 843-22B, the root proteome showed the most substantial variation followed by seed and leaf tissues in response to drought stress (Supplementary Figure S3D). These tissue-specific proteome effects were in contrast to the tolerant genotype (ICTP8203). Considering loadings, the highest positive loading showed proteins with higher abundance in seed and leaf tissues, while negative loading showed proteins with higher abundance in root tissue (Supplementary Figure S3E). Interestingly, it was observed that the seed proteome of both pearl millet genotypes showed only a small difference between control and stress condition.

Contrasting proteome effects were observed in wheat genotypes compared to pearl millet genotypes. Here, the strongest variation was observed in seed and leaf tissues (Supplementary Figure S4A). In TRI 5630, the seed proteome showed the strongest variation in response to drought stress compared to root and leaf tissues (Supplementary Figure S4B). Positive loadings of PC1 represent proteins with higher abundances in seed and leaf tissues, whereas negative loadings depicted higher levels in root tissue (Supplementary Figure S4C and Supplementary Table S13). However, in wheat sensitive genotype White Fife, most substantial variation was observed in the leaf followed by root tissues (Supplementary Figures S4D,E).

## Discussion

### Physiological Comparison of Whole Plant Responses to Drought Stress

**FIGURE 5:**
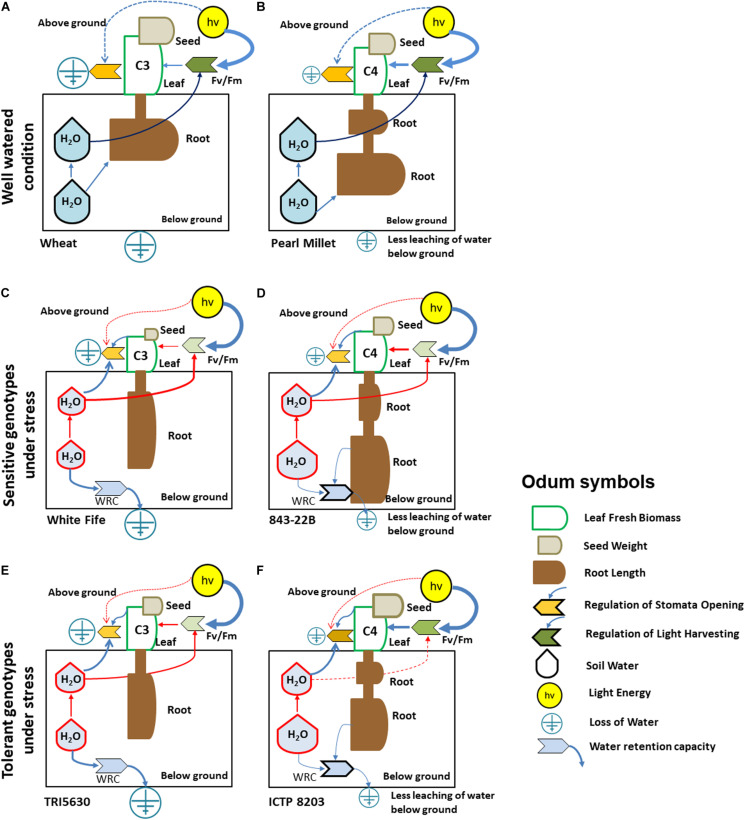
Model for significant and relevant eco-physiological responses of pearl millet and wheat genotypes under drought stress using Odum’s symbols. **(A)** Model for C_3_ wheat under well-watered condition. **(B)** Model for C_4_ pearl millet under well-watered condition. **(C)** Model for White Fife genotype under stress condition. **(D)** Model for 843-22B genotype under stress condition. **(E)** Model for TRI 5630 genotype under stress condition. **(F)** Model for ICTP8203 genotype under stress condition. [Different color code indicates different regulation of the physiological parameters under control (dark color) and stress (light color, diminished regulation; semi-dark color, moderate regulation) condition; Blue line indicates physiological mass/energy transfer; Red line indicates altered physiological response under drought stress; Root length: **(A,B)** feature short, dense roots, **(C,E)** feature moderate/long length and not so dense roots, **(D,F)** exhibit moderate/long and dense roots].

To integrate all physiological information into an intuitive coherent visualization model, we used the visualization strategy of Odum, an approach which integrates systems-theoretical ideas for the analysis of multivariate systems in ecology (Odum, 1994; Weckwerth, 2019). Here, each symbol and size determine systems state variables and their quantity representative for the individual pearl millet and wheat phenotypes under drought stress (Figure 5). Using this visualization strategy an n-dimensional multivariate data matrix and its intrinsic dynamics can be intuitively recognized by visual inspection. This principle is also known as coherent perception, e.g., face recognition, and by using symbols and sizes, we translate highly complex multivariate data into an intuitive visual model otherwise depicted by multivariate statistics such as PCA (Weckwerth and Morgenthal, 2005; Weckwerth, 2008; Weckwerth, 2019). Figures 5A,B determines the physiological response of the wheat and pearl millet under well-watered condition. Grain yield allows direct estimation of the drought tolerance capacity of the individual genotypes (Fischer and Wood, 1979). Under well-watered conditions, pearl millet (843-22B and ICTP8203) and wheat (White Fife and TRI5630) genotypes were found to have comparable grain yields. By contrast, water stress treatment resulted in different grain yields between the genotypes. A significant response was observed between White Fife and TRI5630. White Fife was not able to maintain its yield under drought stress (Figures 5C,E and Supplementary Table S2). A similar response was also observed by Inzanloo and co-workers, where a sensitive genotype Kukri showed a significant drop in the grain yield under stress condition (Izanloo et al., 2008).

In contrast, pearl millet genotypes were able to restore their grain yield under drought stress (Figures 5D,F). Bidinger et al. (1987) reported a similar response of pearl millet under mid-season drought (panicle initiation to flowering) stress. This effect is also related to biomass production during drought stress. The significant reduction in biomass of these drought-tolerant genotypes and the resulting higher harvest index can be attributed as an adaptive response where plants endure low tissue water content through maintenance of cell turgor via osmotic adjustment and cellular elasticity and divert their entire energy to protect seed production under harsh conditions (Farooq et al., 2009). Also at the proteome level, there are relatively small changes in both pearl millet genotypes between well-watered and drought stress conditions which are in stark contrast to wheat. Accordingly, the proteome data reflect the physiological data. In this context, it is being reported by Begg that pearl millet even under favorable conditions tends to have a shorter crop cycle than any other cereals because it has a “built-in” drought escape mechanism of early flowering, inherited from its wild progenitors which are evolved in semi-desert environments. Therefore, pearl millet has not only a short crop cycle but also short grain-filling period and small seed sizes which is a clear advantage in unfavorable growth conditions such as heat and drought (Begg, 1965).

One significant difference in C_3_ and C_4_ plants species is their photosynthetic capacities. Globally, 85% of higher plant species follow C_3_-type photosynthesis whereas only 4% of the plant species belong to the C_4_-type majorly originated in arid regions where high temperature occurs with water stress (Yamori et al., 2014). To investigate these photosynthetic capabilities in more detail chlorophyll content and chlorophyll fluorescence of plants were measured along with stomatal behavior (see below). In all the genotypes, the imposition of drought stress resulted in a decrease in chlorophyll content (Figure 1C and Supplementary Table S2). In this study, the pale leaves with a lower chlorophyll content in White Fife (>10% SPAD units), 843-22B (14% SPAD units) and TRI 5630 (14% SPAD units) senesced early, while the green leaves with a high chlorophyll content in ICTP8203 consistently stayed green. The similar effect was observed in drought-tolerant and susceptible cultivars of peanut under water stress (Katam et al., 2016). Previous studies also revealed that a decrease in chlorophyll concentration under drought stress could be related to the increase in the activity of enzyme chlorophyllase (Ashraf et al., 1994). Drought stress-induced decrease in the pigment content was also previously reported in several plant species, including durum wheat (Loggini et al., 1999). The stay-green trait, in contrast, protects the leaf from the degradation of chlorophyll, stabilizes photosystem and helps to produce normal grain (Thomas and Ougham, 2014; Kamal et al., 2019). However, few reports are available on the mechanism of how this trait protects chlorophyll under drought and which dominant genes control this trait under drought conditions (Walulu et al., 1994). Senescence is typically characterized by chlorophyll loss and a progressive decline in photosynthetic capacity. Early onset of senescence affects assimilation and grain filling in crop plants (Xu et al., 2000). This effect can be correlated to wheat genotypes as they were not able to maintain their chlorophyll content under drought. Accordingly, ICTP8203 showed a stay-green phenotype in contrast to all the other genotypes (Thomas and Howarth, 2000). In this context, F_v_/F_m_ ratio was also highest in ICTP8203 (Figure 5E) followed by 843-22B and TRI 5630 under drought treatment (Figure 1D and Supplementary Table S2). The F_v_/F_m_ ratio of White Fife (Figure 5C) was significantly reduced, indicating a severely impaired photosystem under drought conditions. Programmed leaf senescence is initiated contributing to the plant survival under drought conditions (Lu and Zhang, 1998; Munne-Bosch and Alegre, 2000, 2004; Lu et al., 2002) but also resulting in yield losses (Borrell and Hammer, 2000; Jiang et al., 2004; Rivero et al., 2007).

The tolerant genotypes of pearl millet and wheat ICTP8203 and TRI 5630 also showed a different stomata regulation than the susceptible ones. ICTP8203 and TRI 5630 showed stomatal conductance ranging between 130 and 150 mmol m^–^^2^s^–^^1^ under control condition, already higher than in the sensitive genotypes (White Fife and 843-22B) ranging from 40 to 50 mmol m^–^^2^s^–^^1^. During drought stress, the range of stomata closure was higher and more rapid in the tolerant genotypes. A similar response was observed in the study performed on soybean genotypes (Liu et al., 2005) and *Amaranthus* species under drought stress (Liu and Stutzel, 2002). The rapid stomatal response may act as a drought resistance mechanism, which permits to keep water for later use and thus maintain higher leaf water potentials (Jones, 1974). In principle, stomatal closure protects plants against excessive water loss but also restricts the diffusion of CO_2_ into the photosynthetic parenchyma, especially for C_3_ plants. Stomatal closure causes more significant decrease in transpiration than in photosynthesis rates, thereby increasing the relative leaf water use efficiency (WUE) (Pou et al., 2008). Thus, more dynamic and more extensive regulation of stomata in the tolerant genotypes is one of the pre-requisites for better performance under drought stress. Furthermore, the stomatal limitation on photosynthesis can be accompanied by a decrease in the rate of consumption of ATP and NADPH for CO_2_ assimilation that could result in a decrease in the rate of linear electron transport and consequently in F_v_/F_m_ (Baker and Rosenqvist, 2004) which was primarily observed in the susceptible wheat genotype, White Fife.

Considering the observations of photosynthetic capabilities and stomata regulation the tolerant varieties seem to compensate differences between C_3_- and C_4_-type photosynthesis. The differences are instead found in the stay-green vs. programmed leaf senescence phenotypes.

Another very strong effect is the different root length between wheat and pearl millet but also between the intraspecific genotypes (Figure 5 and Supplementary Figure S5). Due to this difference in root length, water retention capacity is very different for wheat and pearl millet genotypes (Figures 5C–F). Root length was increased in all the genotypes under drought stress (Figures 5C–F). Root length appeared to be an important trait for drought stress tolerance, as reported in the previous study (Leishman and Westoby, 1994). However, unexpectedly, here the sensitive pearl millet genotype 843-22B showed maximum root length as compared to other genotypes. The impact of root system and its mechanism on yield under drought conditions is also comprehensively discussed in many major crops (Tuberosa and Salvi, 2006; de Dorlodot et al., 2007; Comas et al., 2013; Sehgal et al., 2015). Controlled greenhouse and field conditions show different variations of plant functional and molecular traits (Hoehenwarter et al., 2008; Holmgren et al., 2012; Nagler et al., 2018; Weckwerth et al., 2020). In future studies all the drought-related traits and molecular signatures which are described in our study will be also tested under field conditions.

### Proteome Signature for “Stay-Green” and “Senescence” Trait Under Drought Stress

**FIGURE 6:**
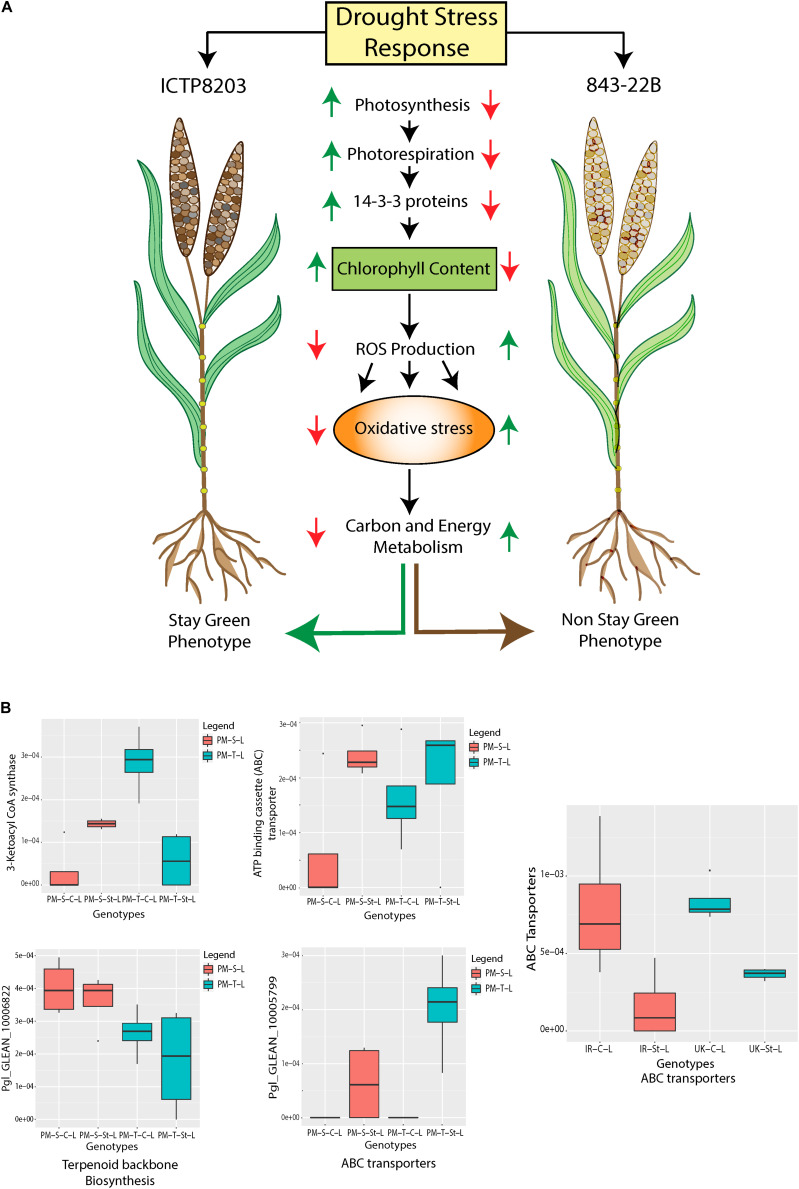
**(A)** Regulation of proteome underlying “Stay-green” trait in pearl millet genotypes (843-22B and ICTP8203). **(B)** Regulation of wax biosynthesis proteins in pearl millet and wheat genotypes under control and stress condition.

Stay-green is an important agronomical trait which can contribute to higher yield production under drought stress condition (Harris et al., 2007; Thomas and Ougham, 2014). However, not much is known about the protein changes leading to this effect. In the present study, a significant change in protein patterns of pearl millet genotypes provided a clear indication of the processes that underlie the stay-green or senescence trait in ICTP8203 and 843-22B, respectively (Figure 6A and Supplementary Table S14). ICTP8203 showed enhanced regulation in photosynthetic activity under drought stress. This correlates with significant higher levels of chlorophyll a-b binding protein (Pgl_GLEAN_10021964), protein kinase (Pgl_GLEAN_10013653), thylakoid lumenal 19 kDa protein (Pgl_GLEAN_10006356), ferredoxin-NADP reductase (Pgl_GLEAN_10033849). This higher photosystem activity resulted in lower levels of reactive oxygen species (ROS) proteins such as peroxidases (Pgl_GLEAN_10014871, Pgl_GLEAN_10027105, Pgl_GLEAN_10006633), glutathione reductase (Pgl_GLEAN_10019381, Pgl_GLEAN_10036180), glutathione synthetase (Pgl_GLEAN_10035689), and peroxiredoxin (Pgl_GLEAN_10024324) under drought stress. Furthermore, the higher levels of several stay-green-associated proteins such as 14-3-3 (Pgl_GLEAN_10007318), chlorophyll synthesis proteins (such as magnesium chelatase ATPase subunit I) (Pgl_GLEAN_10038264), ribulose bisphosphate carboxylase small chain (Pgl_GLEAN_10020566), and uroporphyrinogen decarboxylase (Pgl_GLEAN_10000112) were also observed in ICTP8203. 14-3-3 are the binding proteins that show strong interaction with the enzymes involved in nitrogen and carbon metabolisms which may influence the degradation process (Huber et al., 1996). Overexpression of *Arabidopsis* gene GF14⋌ (which encodes 14-3-3 protein) in cotton lead to stay-green phenotype and also improved drought tolerance of transgenic cotton under drought stress (Yan et al., 2004). Similarly, overexpression of 14-3-3 protein delayed leaf senescence in potato plant (Wilczynski et al., 1998). A protein related to photorespiration (aminomethyltransferase; Pgl_GLEAN_10027187) also showed higher levels in ICTP8203 under drought stress (Supplementary Table S14).

Interestingly, we observed decreased levels of ferrochelatase (Pgl_GLEAN_10011603) which reduces cytotoxicity and in turn, increases chlorophyll biosynthesis (Nagahatenna et al., 2015). In contrast, the sensitive pearl millet genotype 843-22B demonstrated opposite regulation of the proteome compared to ICTP8203, contradicting the stay-green process (Figure 6A). Proteins binned into the functional category of RING finger ubiquitin showed increased levels in 843-22B compared to ICTP8203 under drought stress. The observed regulation of the proteome is positively correlated to the physiological analysis where maximum efficiency of PSII photochemistry (F_v_/F_m_), chlorophyll content and yield was highest in ICTP8203 by the end of the drought stress. Hence, the proteome of ICTP8203 can be identified as “stay-green” signature.

Wheat genotypes demonstrated a different regulation of the proteome compared to pearl millet and did not show the “stay-green” trait at the phenotypic level. The significant changing pattern of proteomes, indicated “senescence” processes (non-sequential and sequential) in TRI5630 and White Fife, respectively (Supplementary Table S14). TRI5630 showed a significant increase in sucrose synthase 4 (SS) (W5I774, A0A1D6SCX5) (more than 6 and 11-fold change, respectively) under drought stress. Increased levels of sucrose content in leaf contributed to non-sequential senescence process. Moreover, increased sugar mobilization promotes stable yield and enhances the tolerance mechanism, which is indicated by higher stem reserve remobilization under drought stress (Shi et al., 2016). Remobilization of stored carbon reserves in wheat is also facilitated under drought stress, which enhances plant senescence process and accelerates grain filling process (Yang et al., 2000, 2001). This can also be correlated to the phenotypic data of TRI5630 showing stable yield despite decreased photosynthetic and chlorophyll biosynthetic proteins.

Interestingly, we also observed increased levels of 4-alpha-glucanotransferase (W5BL76, W5B4C2) (with > 2-fold change) in TRI5630 under drought stress whereas in White Fife these isoforms showed decreased levels or no change under stress. It has been reported that 4-alpha-glucanotransferase is an essential component of the pathway from starch to sucrose and cellular metabolism in leaves at night (Chia et al., 2004). Therefore, increased levels of 4-alpha-glucanotransferase can indicate higher sucrose content in the leaf of TRI5630, which supports the leaf non-sequential senescence process. In White Fife, despite higher levels of photosynthetic and chlorophyll biosynthetic proteins and a decrease in reactive oxygen (ROS) proteins, the total yield was decreased under stress condition (Supplementary Table S14). The possible reason could be a sequential senescence process in which young leaves are successively formed at the top region of the plant and lower older leaves undergo senescence.

### Regulation of Wax Biosynthetic Proteins Under Drought Stress Indicate Higher Capacities for Drought Protective Cuticular Wax Biosynthesis in Pearl Millet Compared to Wheat

Plants are evolved with diverse adaptive strategies to cope with water deficit conditions. Accumulation of cuticular waxes is such a strategy which contributes to drought resistance (Samuels et al., 2008; Lee and Suh, 2015). Seo et al. (2009) demonstrated that increase in the cuticular wax synthesis improves drought tolerance in Arabidopsis species. Reduced wax production leads to drought sensitivity in rice (Zhu and Xiong, 2013). It is also known that cuticular wax biosynthesis is also controlled at post-transcriptional and post-translational levels (Lee and Suh, 2015). At the proteome level, we identified the regulation of two key rate-limiting enzymes of cuticular wax biosynthesis in pearl millet and wheat leaf tissue which includes 3-ketoacyl-CoA synthase (KCS) and ATP-binding cassette (ABC) transporter. In pearl millet genotypes, one of the 3-ketoacyl-CoA synthase (Pgl_GLEAN_10030730) showed enhanced levels under stress condition in the sensitive genotype 843-22B compared to the tolerant genotype ICTP8203 (Figure 6B). Similarly, GWAS study lead to the identification 3 SNPs located between two predicted genes encoding for 3-ketoacyl-CoA synthase in pearl millet under drought stress (Debieu et al., 2018). Interestingly in wheat genotypes (White Fife and TRI 5630), this gene family was not detected in either condition. 3-ketoacyl-CoA synthase is not only involved in decarbonylation and acyl-reduction of wax synthesis pathways but also involved in elongation of C24 fatty acids which is an essential condensation step during wax and suberin biosynthesis. A study performed by Yu and coworkers demonstrated that OsWSL1 encodes 3-ketoacyl-CoA synthase (KCS) genes in rice, catalyzes the formation of C20–C24 VLCFA precursors of leaf waxes. The OsWSL1 mutant showed a pleiotropic phenotype with decreased growth, sparse wax crystals and drought sensitivity, suggesting that OsWSL1 may be relevant to drought tolerance (Yu et al., 2008). Export of cuticular wax is mediated by the ATP binding cassette (ABC) transporters (Pighin et al., 2004; Bird et al., 2007; Panikashvili et al., 2007). In both the pearl millet genotypes three ATP-binding cassette (ABC) transporter proteins (Pgl_GLEAN_10004859, Pgl_GLEAN_10002141, Pgl_GLEAN_10006800) were identified and showed increased levels under stress condition compared to controls. Intriguingly, this protein showed an opposite regulation pattern in wheat genotypes (A0A1D5VIG5, A0A1D6C5F5, A0A1D6BMJ3, A0A1D6D783) under drought stress (Figure 6B). Information on wax biosynthetic genes is sparse in wheat due to the lack of functional genomic studies. However, it is known that wheat employs another parallel wax biosynthetic pathway, which is predominant in the reproductive stages and responsible for the biosynthesis of β-diketones (Tulloch, 1973). Recently, the pearl millet genome study has shown substantial enrichment and expansion of wax biosynthetic genes which may contribute to heat and drought tolerance of this crop in semi-arid regions (Varshney et al., 2017) compared to other cereals. Taking a look at the translational level of these genes in the leaf tissue of pearl millet genotypes, four genes Pgl_GLEAN_10006822, Pgl_GLEAN_10030730, Pgl_GLEAN_10005799, and Pgl_GLEAN_10005798 were identified which belonged to the group of terpenoid backbone biosynthesis, suberin biosynthesis and ABC transporters, respectively. These data indicate that wax biosynthesis is enhanced in pearl millet at the proteome level, especially in ICTP8203. In contrast, these pathways are not detected in wheat and drought-dependent enhancement is also not observed.

### Drought Responsive Regulation of the Key Photosynthetic Proteins of Pearl Millet (NAD-ME Type) and Wheat Under Drought Stress

**FIGURE 7:**
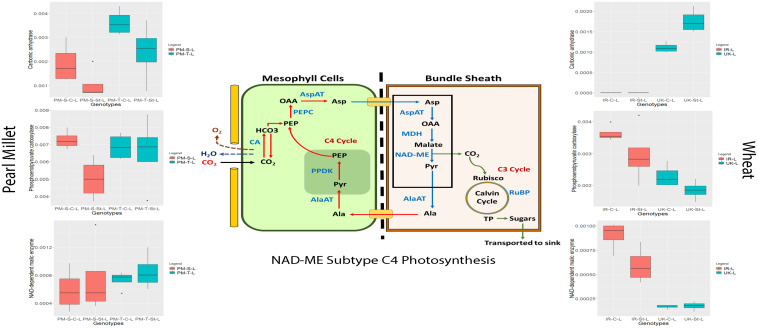
Regulation of NAD-ME subtype C_4_ photosynthesis proteins in pearl millet and wheat genotypes under control and stress condition (Ala, Alanine; Asp, Aspartate; Mal, Malate; Pyr, Pyruvate; OAA, Oxaloacetate; PEP, Phosphoenolpyruvate; CA, Carbonic Anhydrase; PEPC, Phosphoenolpyruvate Carboxylase; PPDK, Pyruvate Phosphate Dikinase; AspAT, Aspartate Aminotransferase; AlaAT, Alanine Aminotransferase; MDH, Malate Dehydrogenase; NAD-ME, Nicotinamide adenine dinucleotide-dependent Malic Enzyme; Rubisco, Ribulose 1,5-bisphosphate Oxygenase-Carboxylase; TP, Triose Phosphate, RuBP, Ribulose 1,5-bisphosphate; HCO_3_, Bicarbonate; CO_2_, Carbon dioxide; H_2_O, Water).

Global depletion of atmospheric CO_2_ levels led to the evolution of C_4_ photosynthesis from ancestral C_3_ photosynthesis. Among C_4_ plants, there are three biochemical subtypes, based on the C_4_ acid decarboxylation enzyme in the bundle sheath (Hatch, 1987; Leegood, 2002) (1) NADP-malic enzyme (NADP-ME) type, (2) NAD-malic enzyme (NAD-ME) type, and (3) phosphoenolpyruvate carboxykinase (PEPCK) type (Leegood, 2002). Distribution of C_4_ grasses is strongly influenced by rainfall level, e.g., areas with decreasing rainfall (from 900 to 50 mm per annum) demonstrates an increased abundance of NAD-ME subtype grasses compared to NADP-ME subtype grasses. This geographical distribution of C_4_ grasses with different biochemical subtypes may also reflect different drought tolerance capacities (Ghannoum, 2009). However, at the proteome level, there is no evidence suggesting that these three C_4_ biochemical pathways have different sensitivities to water stress. Pearl millet has been classified as a NAD-ME subtype (Edwards and Walker, 1983). The enzymes involved in C_4_ photosynthesis are also present in C_3_ plants of course without the Kranz anatomy but expression regulation is different, and activities are much lower. These enzymes operate for different metabolic processes, and they also have different inter- and intracellular localization. At the proteome level, we have identified all significant enzymes associated with the C_4_ pearl millet NAD-ME subtype photosynthesis under control and drought stress in both the genotypes wheat with different abundance level (Figure 7 and Supplementary Figure S6).

Carbonic anhydrase (CA) and phosphoenolpyruvate carboxylase (PEPC) are two important enzymes at the beginning of the C_4_ carbon fixation process and may be directly related to photosynthesis efficiency. They both are located in the cytosol of mesophyll cells, whereas in C_3_ plants, they are predominantly located in chloroplast stroma (Ignatova et al., 2019). CA at the proteome level showed decreased levels under stress condition compared to controls in both the genotypes of pearl millet (Pgl_GLEAN_10019649, Pgl_GLEAN_10007313, Pgl_GLEAN_10019645). In contrast, PEPC (Pgl_GLEAN_10033512, Pgl_GLEAN_10037989, Pgl_GLEAN_10033055, Pgl_GLEAN_10026714, Pgl_GLEAN_ 10036281) showed increased levels under drought stress in the tolerant genotype ICTP8203 (Figure 7). In wheat genotypes, CA (A0A1D5WHU7) showed increased levels in White Fife under stress condition but did not show any regulation in TRI 5630. PEPC (A0A1D5YFR1, A0A1D6RUC3, A0A1D6C446, A0A1D6BRX9, A0A1D6RR40, A0A1D6ALV9, A0A1D5WD42, and A0A1D6AHU2) showed decreased levels in both the genotype under stress condition (Figure 7). Phosphoenolpyruvate carboxylase (PEPC), the second most abundant enzyme of the C_4_ pathway, is an important and multifaceted enzyme that catalyzes the irreversible β-carboxylation of phosphoenolpyruvate (PEP) to yield oxaloacetate (OAA) and inorganic phosphate (Pi) (Hatch, 1987). It also provides oxaloacetic acid to the tricarboxylic acid cycle (TCA cycle) and catalyzes reactions involved in amino acid metabolism in C_3_ plants and non-photosynthetic tissues of C_4_ plants (Westhoff and Gowik, 2004). Ding and co-workers demonstrated that overexpression of PEPC led to higher CO_2_ assimilation compared to wild-type under progressive drought stress (Ding et al., 2015). Similarly, Jiao et al. (2002) determined that expression of maize C_4_-PEPC in transgenic rice led to improved photodetoxication and photosynthetic capacity under drought stress. However, mutation of two PEPC genes of Arabidopsis [PPC1 (AT1G53310), PPC2 (AT2G42600)] led to severe growth arrest phenotype and reduced the synthesis of malate and citrate and severely suppressed ammonium assimilation (Shi et al., 2015).

Interestingly, NAD-malic enzyme (NAD-ME) showed increased levels in stressed plants compared to controls in both the genotypes of pearl millet (Pgl_GLEAN_10013928, Pgl_GLEAN_10034558). In contrast, the reverse trend was observed in wheat genotypes (A0A1D5S6L5, A0A1D5U9X7) under drought stress (Figure 7). NAD-ME plays a major role in determining flux through the TCA cycle by providing pyruvate for oxidation. However, studies claim that antisense potato lines do now show any perturbation in flux through the TCA cycle but an alteration in glycolytic metabolism (Jenner et al., 2001).

Several other enzymes were also identified which included malate dehydrogenase (MDH), pyruvate phosphate dikinase (PPDK), aspartate aminotransferases which are majorly located in mitochondria of bundle sheath and cytosol of mesophyll cells in C_4_ plants (Taniguchi et al., 1995), and alanine aminotransferases that lead to the reversible conversion of pyruvate to alanine. All the identified proteins showed reduced levels under stress condition compared to controls in pearl millet genotypes with a significant response in 843-22B (Supplementary Figure S6). In contrast, in wheat genotypes, only the sensitive genotype White Fife showed decreased levels of these proteins compared to controls. In contrast, the tolerant genotype TRI 5630 showed enhanced regulation under stress condition. These identifications indicate that the combined activities of several proteins may enable tolerant genotypes ICTP8203 and TRI 5630 to retain its photosynthetic efficiency under drought stress compared to sensitive genotypes 843-22B and White Fife. However, the pattern does not lead to a clear functional interpretation, and future studies will focus more on these pathways.

### Integration of Proteome With Physiological Data of Pearl Millet and Wheat Phenotypes Demonstrates the Enormous Plasticity of Drought Stress Adaptation

The physiological data of the preceding discussions were used as predictable traits to identify protein networks which show high predictive power. Accordingly, we had several scenarios where we could exploit the predictive power of protein correlation networks for traits such as yield (seed weight), harvest index, root length and many more. For statistical modeling, we employed a method called sparse least square discriminant analysis sPLS-DA (see section “Materials and Methods”). Plants use different strategies to cope with drought stress, ranging from drought avoidance to desiccation. Drought avoidance is associated with the minimization of water loss and simultaneously maximization of water uptake (Ludlow and Muchow, 1990). Evidence suggests that the adverse effects of drought can be successfully avoided by changing carbon allocation patterns to allow the formation of a deep root system before the onset of a growth-limiting water shortage. Accordingly, root traits are now considered as important targets under drought stress for yield improvement. Therefore, we decided to identify protein correlation networks predictive for root length which has been highly distinguishable between pearl millet and wheat genotypes. Pearl millet genotypes showed increased root length under stress condition compared to controls with extensive elongation in 843-22B. Using sPLS, key stress proteins correlating with the root length were identified for each genotype individually (Supplementary Figure S7 and Supplementary Table S15). The identified proteins were functionally classified using MAPMAN and represented in an interacting network based on the color of the functional bins (Supplementary Table S15).

**FIGURE 8:**
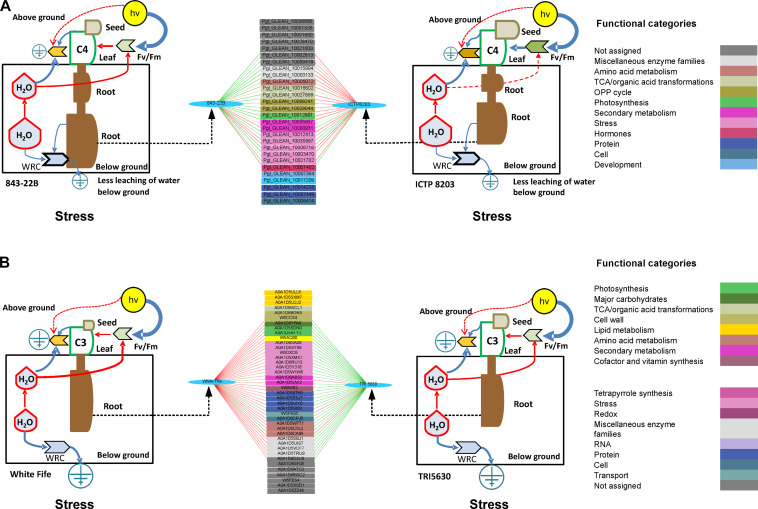
**(A)** Discriminant analysis determine interactive network using quantified proteins as a predictor and root length as a factor in 843-22B and ICTP8203 under stress condition and correlated with the odum’s model of physiology. **(B)** Discriminant analysis determine interactive network using quantified proteins as a predictor and root length as a factor in White Fife and TRI 5630 under stress condition and correlated with the odum’s model of physiology. Only those correlations equal or higher, in absolute value, than 0.9 are shown. Green line = negative correlation, Red line = positive correlation.

Furthermore, the discriminant analysis allowed determining differences of predictive protein levels between 843-22B and ICTP8203 for root length (Figure 8A). The genotypes showed remarkable plasticity and differences in the same set of predictive protein correlation networks (Figure 8A). Based on this analysis, proteins binned in the functional categories of stress, reactive oxygen species (ROS) and oxidative pentose phosphate pathway (OPP) showed the highest correlation scores with the root length. Annexin protein was positively correlated to the 843-22B (Figure 8A). Plant annexins are Ca^2^^+^ dependent phospholipid-binding proteins, and they participate in the regulation of plant development as well as in plant protection from the drought and other stresses. Annexins have been identified as a component of signal-transduction pathways in many species, such as soybean (Feng et al., 2013) and rice (Jami et al., 2012). In the study performed by Konopka-Postupolska and Clark, overexpression of annexin 1 (AnnAt1) not only allowed plants to retain their growth and productivity potentials under severe drought stress condition but also provided them protection against oxidative stress, however the mechanism of protection mediated by AnnAt1 can be different in different tissues (Konopka-Postupolska and Clark, 2017). Similarly, defense-related proteins such as diseases resistance response protein also showed positive correlation scores with increased root growth of 843-22B (Figure 8A) indicating activation of defense-related pathways.

Two isoforms of phenylalanine ammonia-lyase showed a positive correlation with the tolerant ICTP8203 root phenotypes (Figure 8A) suggesting an activated biosynthesis of antioxidative compounds. Phenylpropanoid compounds not only fulfill various essential functions during plant development but also they act as essential protectants against various biotic and abiotic environmental stresses. Two isoforms of peroxidases also showed a positive correlation with the ICTP8203 root phenotype. Peroxidases are the bifunctional enzymes which not only act on oxidizing agents but also produce ROS (Passardi et al., 2004). In plants, this enzyme is involved in many physiological and developmental processes which include their association with cell elongation processes, and also with reactions that restrict growth. Reduction in cell wall plasticity leads to the stiffening of the cell wall. Covalently bound cell wall peroxidases play a significant role in this process either by catalyzing the polymerization of the phenolic monomers of lignin or by participating in the formation of cross-bridges between various polysaccharide polymers. Based on the identified protein candidates, it can be concluded that under drought stress, antioxidant activity was enhanced in ICTP8203 compared to 843-22B.

The same approach was applied to the stress proteome of the wheat genotypes and correlated to the root phenotypes (Supplementary Figure S8 and Supplementary Table S16). This analysis revealed rather different patterns compared to pearl millet except peroxidases, which were also identified. In the discriminant analysis, proteins majorly binned in the functional category of lipid metabolism, stress, amino acid metabolism, carbohydrate metabolism and secondary metabolism were determined (Figure 8B). In the physiological analysis of White Fife and TRI 5630 showed increased root length under stress condition compared to controls. Two isoforms of eukaryotic translation initiation factor 3 (eIF3) showed a positive correlation with White Fife (Figure 8B). A study performed by Singh and coworker demonstrated that the overexpression of a gene encoding eIF3g (TaeIF3g: *Triticum aestivum* eukaryotic initiation factors), one of the 11 subunits of eukaryotic translation initiation factor 3 (eIF3), showed enhanced tolerance to abiotic stress in yeast and transgenic lines of Arabidopsis (Singh et al., 2013). β-glucosidase is actively involved in cell-wall modification, stress defense, phytohormone signaling, and secondary metabolism; it also plays an essential role in the hydrolysis of cellulose by converting cellobiose to glucose. Here in this study, β-glucosidase and β-amylase showed a positive correlation with White Fife, which indicates that carbohydrate metabolism was activated in the roots of White Fife under drought stress condition. In soybean roots, β-glycosidase showed higher accumulation under stress condition (Wang and Komatsu, 2018).

**FIGURE 9:**
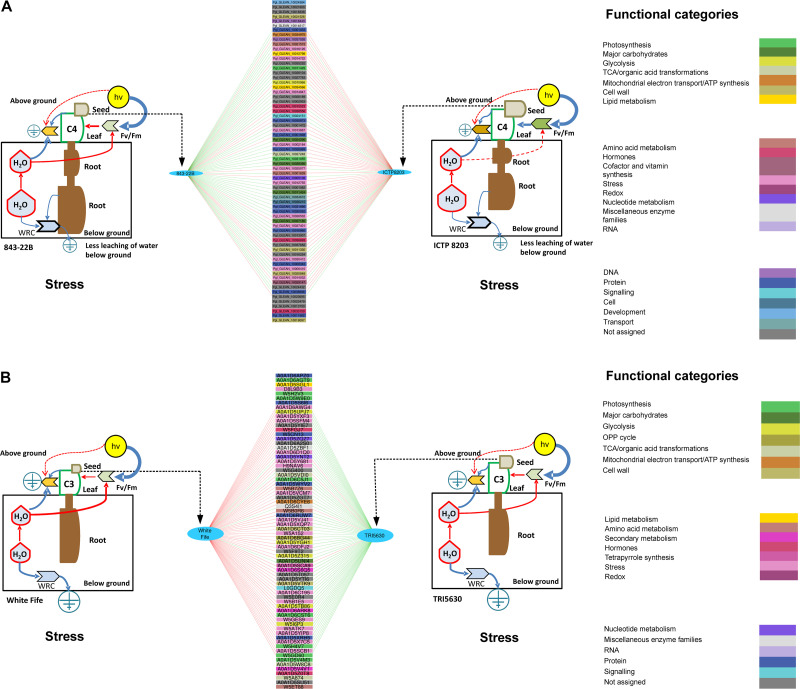
**(A)** Discriminant analysis determine interactive network using quantified proteins as a predictor and seed weight as a factor in 843-22B and ICTP8203 under stress condition and correlated with the odum’s model of physiology. **(B)** Discriminant analysis determine interactive network using quantified proteins as a predictor and seed weight as a factor in White Fife and TRI 5630 under stress condition and correlated with the odum’s model of physiology. Only those correlations equal or higher, in absolute value, than 0.9 are shown. Green line = negative correlation, Red line = positive correlation.

Drought stress has adverse effects on seed production; however, it also largely depends upon the duration of drought stress, the growth stage of the plant and seed filling period. In the present study, genotypes of wheat and pearl millet showed contrasting effects of drought on seed productivity. Based on the physiological analysis, pearl millet genotypes were able to maintain productivity under stress conditions compared to the wheat genotypes, especially ICTP8203. The stress proteome of pearl millet genotypes was correlated with the seed weight (Supplementary Figure S9 and Supplementary Table S15) and proteins binned in the functional category of stress, carbohydrate metabolism, lipid metabolism, amino acid metabolism, signaling and development were identified (Figure 9A; Supplementary Table S15). Two isoforms of late embryogenesis abundant (LEA) protein and lipid transfer protein (LTP) were identified and showed a positive correlation with ICTP8203. These proteins were also differentially abundant in seed proteome of ICTP8203 and not in 843-22B, which confer that these proteins can be used as potential tissue-specific genotype marker for ICTP8203 (Figure 9A). Glycogen synthase which is binned in the functional category of carbohydrate metabolism also showed a positive correlation with ICTP8203, and it has been reported that glycogen synthase confers enhanced tolerance against abiotic stresses (Joshi et al., 2018; Figure 9A). Similarly, two isoforms of heat shock proteins were also identified, showing a positive correlation with ICTP8203. Many small heat shock proteins (sHSPs) play a major role in the protection of seeds from desiccation. This also indicates that accumulation of protective proteins might play a significant role in the drought-responsive mechanism of ICTP8203, leading to healthy seeds even under water deficit condition; indeed, the productivity of plants does not get as severely affected as for other compared genotypes under drought stress. Further, the chitinase protein was identified, which showed a positive correlation with 843-22B (Figure 9A). In plants, chitinases are induced in defense response against abiotic stress and constitutively expressed in plant organs, such as seeds. A class 1 chitinase has been previously identified as an abundant protein in the soybean (*Glycine max*) mature seed coat (Gijzen et al., 2001).

sPLS and discriminant analysis of wheat seed proteome lead us to identify proteins binned in the functional category of reactive oxygen species (ROS), photosynthesis, stress, glycolysis, and secondary metabolism (Figure 9B, Supplementary Figure S10, and Supplementary Table S16). Several isoforms of histones were identified, which showed a positive correlation with White Fife. Increasing evidence shows that chromatin organization plays a very important role in the transcriptional reprogramming of stress-responsive gene expression, proper resource allocation to growth vs. stress responses, acclimation, and long-term stress memory (Chinnusamy and Zhu, 2009; Mirouze and Paszkowski, 2011). Several studies have also reported that histones play an important role during the process of grain filling and drought stress leads to the modification of this protein (Kalamajka et al., 2010). Further, two isoforms of superoxide dismutase were identified, which showed a positive correlation with White Fife, indicating that scavenging mechanism was activated under drought stress. Similarly, two isoforms of phosphoglycerate kinase also showed a positive correlation with White Fife. Phosphoglycerate kinase is an enzyme that catalyzes the reversible transfer of a phosphate group from 1, 3-bisphosphoglycerate (1, 3-BPG) to ADP producing 3- phosphoglycerate (3-PG) and ATP (Fermo et al., 2012). These results indicate that energy metabolism was activated under drought stress.

## Conclusion

The need for drought-tolerant crops is critical, and will surely grow in coming years because of the increase in food demand per capita, ongoing degradation of soil, depletion of water resources, and the accelerating effects of global climate change. Therefore, the development of improved drought-tolerant varieties is the challenge for plant breeders and crop physiologists. One strategy to increase crop productivity is to endow them with C_4_ traits. However, this approach needs a better understanding of the molecular and physiological level. Here, we exploited a comparative approach to define molecular and physiological components of C_3_ and C_4_ plants under drought stress and identified the traits which can be of help in the ongoing engineering process. The results herein presented, reflect the complex mechanism at physiological and proteome level that C_3_ wheat and C_4_ pearl millet employ in adapting to the drought stress environment, which also testifies toward the plasticity of these plants. An intriguing result is that tolerant lines of pearl millet and wheat seem to override already differences between C_3_- and C_4_-type photosynthesis. These results are subject to future studies. Our results also provide evidence that one of the most substantial advantages for enhanced drought-tolerance is the stay-green trait, and we demonstrate the first proteome signature in pearl millet for this trait. Other important characteristics of drought resistance are related to root morphology, efficient photosynthetic machinery and wax biosynthetic enzymes.

We suggest several strategies for engineering enhanced tolerance in the crop plants under drought stress: (1) Identification and mapping of quantitative trait loci (QTL) for root length in cereal crops as it is an important trait for survival under drought stress. (2) Enrichment and expansion of stay-green protein signatures and wax biosynthetic genes. (3) Further investigation of reactive oxygen species (ROS) in roots as they can act as efficient signaling molecules which can enhance root to shoot crosstalk under drought stress. This study also provides information on yield-associated traits, tissue and genotype specific marker that can be exploited for marker-assisted breeding for improving drought tolerance in crop plants. This knowledge is very important because of the large discrepancy between gene expression level and protein activities, which are dynamically modified by actual field conditions in a strongly fluctuating climate. To our knowledge, it is the first report on a comparative physiological and proteomic analysis of wheat and pearl millet in response to drought stress, thus, serving as a large-scale reference study for future investigations.

## Data Availability Statement

The datasets presented in this study can be found in online repositories. The names of the repository/repositories and accession number(s) can be found in the article/Supplementary Material.

## Author Contributions

PC, AG, and WW conceived and designed the experiments and wrote the manuscript. AG, PC, and GB performed the experiments. PC, AG, GB, LV, ŽR, MB, PB, SJ, WL, XS, KG, RKV, and WW analyzed the data. All authors contributed to editing and agreed on the final version.

## Conflict of Interest

The authors declare that the research was conducted in the absence of any commercial or financial relationships that could be construed as a potential conflict of interest.

## Footnotes

1 http://genevenn.sourceforge.net/

## Supplementary Material

Supplementary Figure 1 **(A)** Orthogonal partial least squares discriminant analysis (OPLS–DA) was performed considering all the factors and variables of physiological parameters under control and stress condition in pearl millet and wheat genotypes. **(B)** Principal component analysis (PCA) of all the physiological parameters under control and stress in pearl millet and wheat genotypes.

Supplementary Figure 2 Venn diagrams represent total proteome identification in root, leaf and seed tissues of pearl millet and wheat genotypes under control and stress condition.

Supplementary Figure 3 Principal component analysis (PCA) of pearl millet genotypes. **(A)** PCA considering all the tissues of pearl millet tolerant and sensitive genotypes (84322B and ICTP8203) under control and stress. **(B)** PCA of ICTP8203 under control and drought stress in roots, seeds, and leaves. **(C)** Loading graphs of average for 100 positive and 100 negative loadings. **(D)** PCA of 843-22B under control and drought stress in roots, seeds, and leaves; **(E)** Loading graphs of average for 100 positive and 100 negative loadings.

Supplementary Figure 4 Principal component analysis (PCA) of wheat genotypes. **(A)** PCA considering all the tissues of wheat tolerant and sensitive genotypes (White Fife and TRI 5630) under control and stress. **(B)** PCA of TRI 5630 under control and drought stress in roots, seeds, and leaves. **(C)** Loading graphs of average for 100 positive and 100 negative loadings. **(D)** PCA of White Fife under control and drought stress in roots, seeds, and leaves. **(E)** Loading graphs of average for 100 positive and 100 negative loadings.

Supplementary Figure 5 Root length measurements: Box plots represent root length of the plants under control and stress condition in pearl millet and wheat genotypes; Root length pictures of pearl millet and wheat plants under control and stress condition.

Supplementary Figure 6 Regulation of C_4_ photosynthesis pathway proteins in pearl millet (843-22B and ICTP8203) and wheat (White Fife and TRI 5630) genotypes under control and stress condition.

Supplementary Figure 7 sPLS-based network constructed using quantified proteins as a predictor and root length as a factor for pearl millet genotypes (843-22B and ICTP8203) under stress condition. The interacting network was correlated with the odum’s model of physiology. Only those correlations equal or higher, in absolute value, than 0.9 are shown. Green line = negative correlation, Red line = positive correlation.

Supplementary Figure 8 sPLS-based network constructed using quantified proteins as a predictor and root length as factor for wheat genotypes (White Fife and TRI5630) under stress condition. The interacting network was correlated with the odum’s model of physiology. Only those correlations equal or higher, in absolute value, than 0.9 are shown. Green line = negative correlation, Red line = positive correlation.

Supplementary Figure 9 sPLS-based network constructed using quantified proteins as a predictor and seed weight as factor for pearl millet genotypes (84322B and ICTP8203) under stress condition. Interacting network was correlated with the odum’s model of physiology. Only those correlations equal or higher, in absolute value, than 0.9 are shown. Green line = negative correlation, Red line = positive correlation.

Supplementary Figure 10 sPLS-based network constructed using quantified proteins as a predictor and seed weight as factor for wheat genotypes (White Fife and TRI 5630) under stress condition. The interacting network was correlated with the odum’s model of physiology. Only those correlations equal or higher, in absolute value, than 0.9 are shown. Green line = negative correlation, Red line = positive correlation.

Supplementary Table 1 Details of the sample code for the raw files measured and submitted in PRIDE repository for Wheat and Pearl millet genotypes.

Supplementary Table 2 Physiological parameters under control and drought stress in pearl millet and wheat genotypes.

Supplementary Table 3 Qualitative and quantitative identification of proteins in pearl millet (843-22B and ICTP8203) genotypes.

Supplementary Table 4 Qualitative and quantitative identification of proteins in wheat (White Fife and TRI5630) genotypes.

Supplementary Table 5 Two-way ANOVA analysis of pearl millet (843-22B; ICTP8203) and wheat (White Fife; TRI5630) genotypes.

Supplementary Table 6 Differentially expressed proteins in root, leaf, seed tissues of pearl millet sensitive (843-22B) and tolerant (ICTP8203) genotypes.

Supplementary Table 7 Differentially expressed proteins in root, leaf, seed tissues of wheat sensitive (White Fife) and tolerant (TRI 5630) genotypes.

Supplementary Table 8 Cluster analysis of pearl millet (843-22B and ICTP8203) genotypes using Kmeans in Matlab.

Supplementary Table 9 Cluster analysis of wheat (White Fife and TRI5630) genotypes using Kmeans in Matlab.

Supplementary Table 10 Functional categorization of the identified proteins in pearl millet (843-22B and ICTP8203) genotypes.

Supplementary Table 11 Functional categorization of the identified proteins in wheat (White Fife and TRI5630) genotypes.

Supplementary Table 12 PCA loadings of pearl millet genotypes (843-22B and ICTP8203).

Supplementary Table 13 PCA loadings of wheat genotypes (White Fife and TRI5630).

Supplementary Table 14 Proteome regulation underlying ‘Stay-green’ and “Senescence” trait in pearl millet genotypes (843-22B and ICTP8203) and wheat genotypes (White Fife and TRI5630).

Supplementary Table 15 sPLS and discriminant network analysis, pearl millet (843-22B and ICTP8203) stress proteins integrated with root length and seed weight and color-code-bins used for the functional category for network analysis.

Supplementary Table 16 sPLS and discriminant network analysis, wheat stress (White Fife and TRI5630) proteins integrated with root length and seed weight.
