# Supplementary material for: Physiological and Proteomic Signatures Reveal Mechanisms of Superior Drought Resilience in Pearl Millet Compared to Wheat
Source: Front Plant Sci. 2021 Jan 13;11:600278. doi: 10.3389/fpls.2020.600278 (PMC7838129; doi:10.3389/fpls.2020.600278)
Supplement: Supplementary Figure 1 — (A) Orthogonal partial least squares discriminant analysis (OPLS–DA) was performed considering all the factors and variables of physiological parameters under control and stress condition in pearl millet and wheat genotypes. (B) Principal component analysis (PCA) of all the physiological parameters under control and stress in pearl millet and wheat genotypes. [file Data_Sheet_1.PDF]

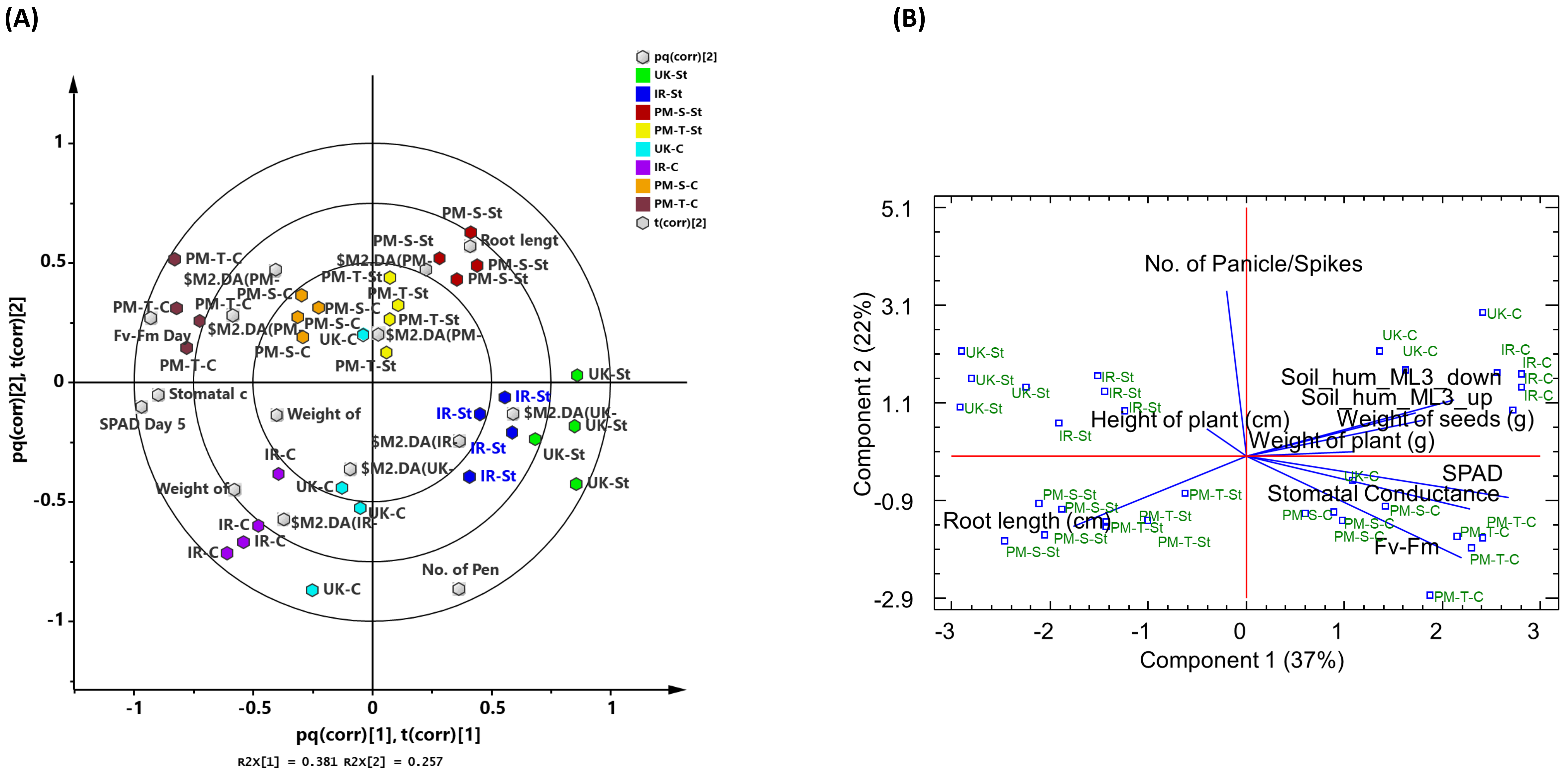
(A)
pq(corr)[2]
UK-St
IR-St
PM-S-St
PM-T-St
UK-C
IR-C
PM-S-C
PM-T-C
t(corr)[2]
pq(corr)[2], t(corr)[2]
pq(corr)[1], t(corr)[1]
R2X[1] = 0.381 R2X[2] = 0.257
Root lengt
Fv-Fm Day
Stomatal c
SPAD Day 5
Weight of
No. of Pen
(B)
No. of Panicle/Spikes
Soil_hum_ML3_down
Soil_hum_ML3_up
Height of plant (cm)
Weight of seeds (g)
Weight of plant (g)
SPAD
Stomatal Conductance
Root length (cm)
Fv-Fm
Component 1 (37%)
Component 2 (22%)

Figure S1

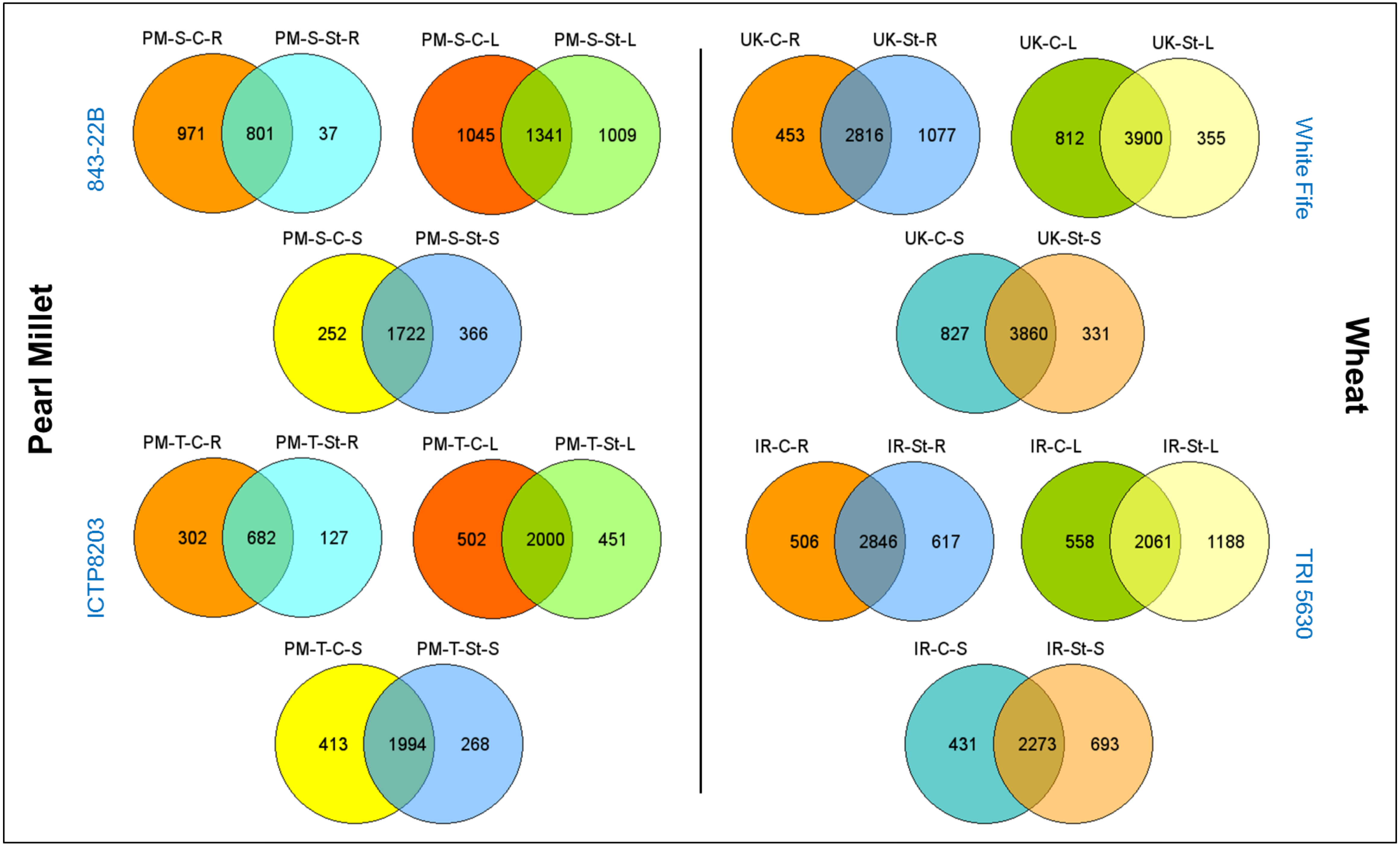
Pearl Millet
843-22B
PM-S-C-R
PM-S-St-R
971
801
37
PM-S-C-L
PM-S-St-L
1045
1341
1009
PM-S-C-S
PM-S-St-S
252
1722
366
ICTP8203
PM-T-C-R
PM-T-St-R
302
682
127
PM-T-C-L
PM-T-St-L
502
2000
451
PM-T-C-S
PM-T-St-S
413
1994
268
Wheat
White Fife
UK-C-R
UK-St-R
453
2816
1077
UK-C-L
UK-St-L
812
3900
355
UK-C-S
UK-St-S
827
3860
331
TRI 5630
IR-C-R
IR-St-R
506
2846
617
IR-C-L
IR-St-L
558
2061
1188
IR-C-S
IR-St-S
431
2273
693

Figure S2

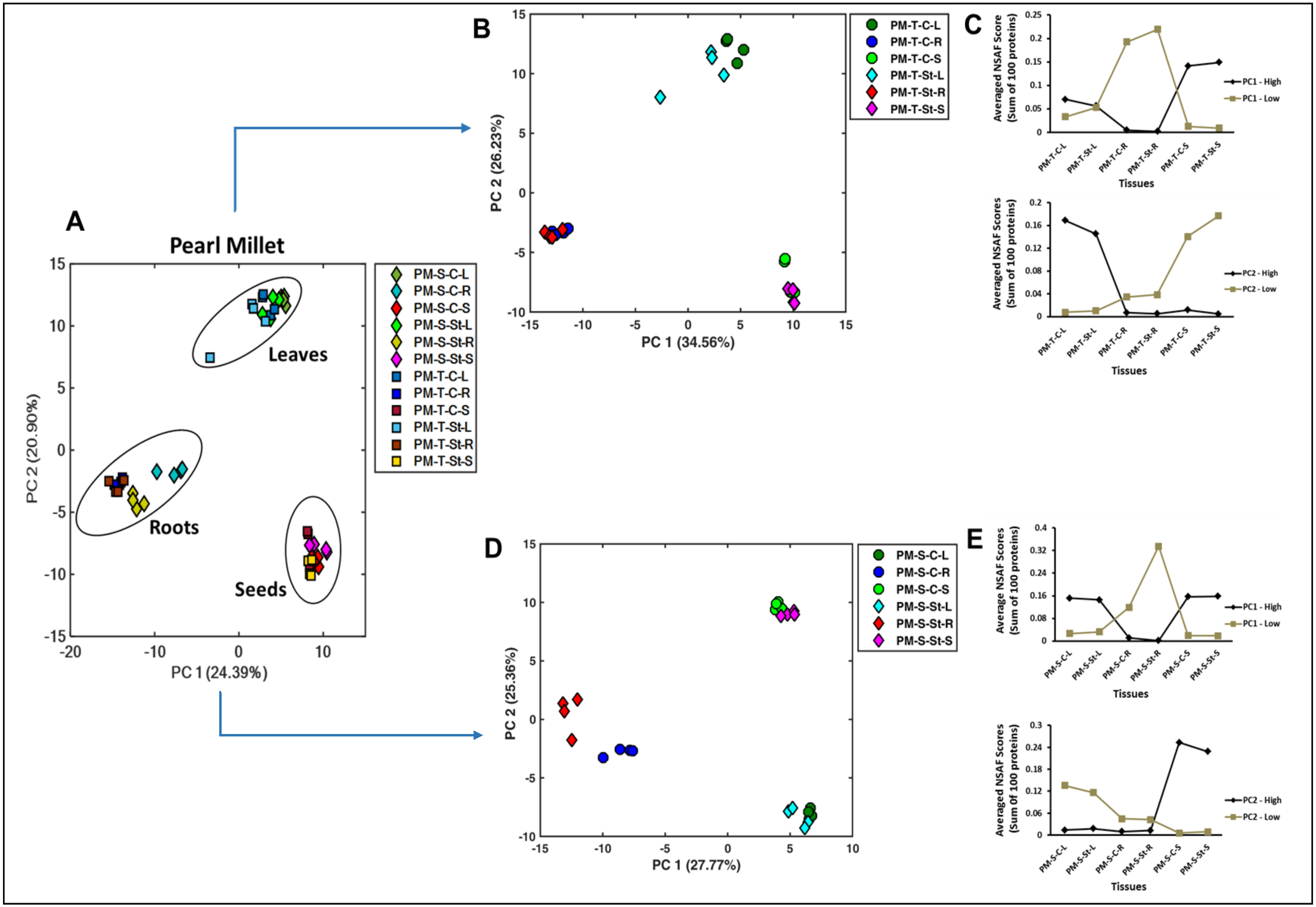
A
Pearl Millet
Leaves
Roots
Seeds
PC 1 (24.39%)
PC 2 (20.90%)
PM-S-C-L
PM-S-C-R
PM-S-C-S
PM-S-St-L
PM-S-St-R
PM-S-St-S
PM-T-C-L
PM-T-C-R
PM-T-C-S
PM-T-St-L
PM-T-St-R
PM-T-St-S
B
PC 1 (34.56%)
PC 2 (26.23%)
C
Averaged NSAF Score (Sum of 100 proteins)
PC1 - High
PC1 - Low
PC2 - High
PC2 - Low
Tissues
D
PC 1 (27.77%)
PC 2 (25.36%)
E
Average NSAF Scores (Sum of 100 proteins)
Averaged NSAF Scores (Sum Of 100 proteins)

Figure S3

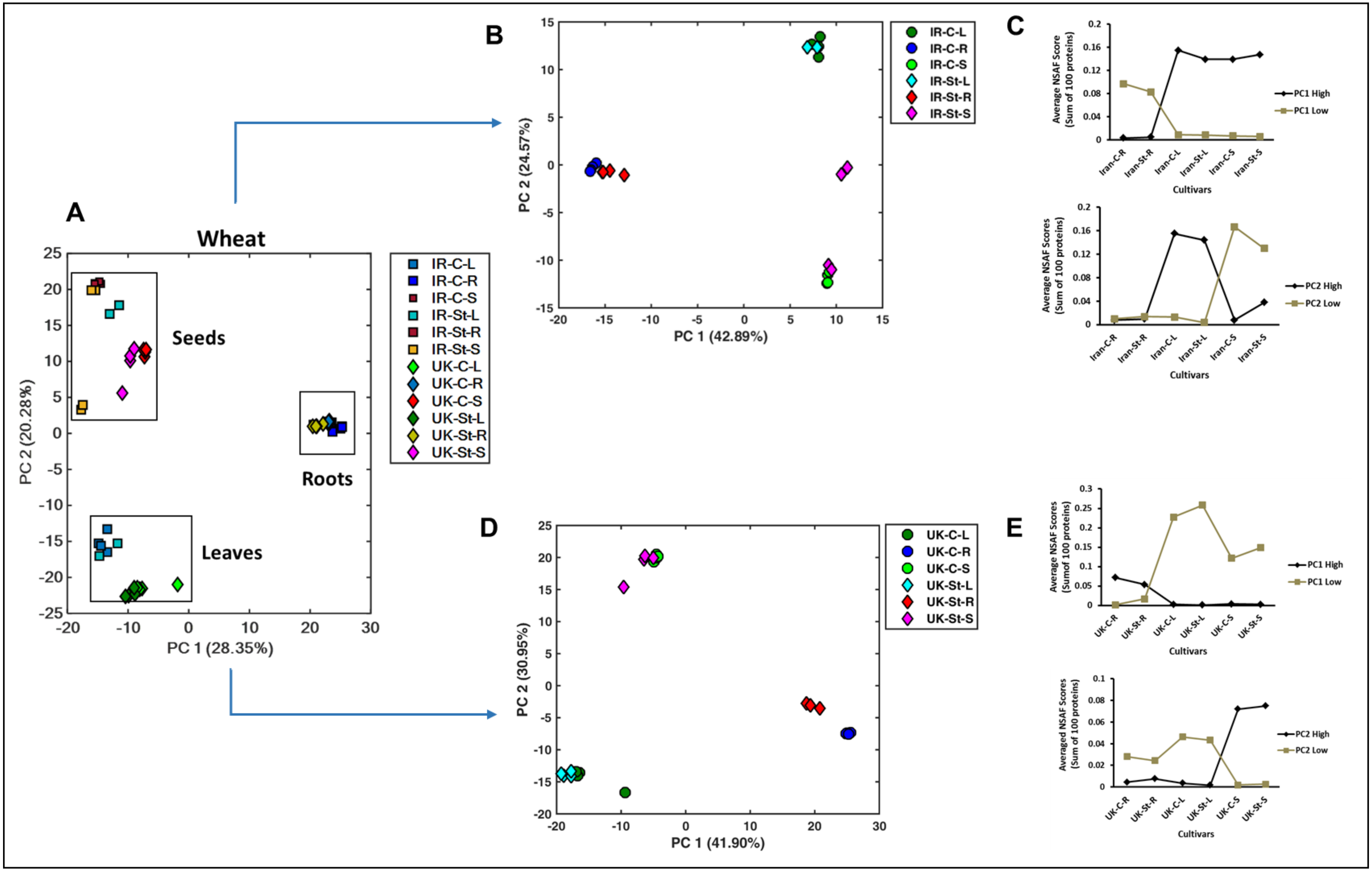
A
Wheat
Seeds
Roots
Leaves
PC 1 (28.35%)
PC 2 (20.28%)
IR-C-L
IR-C-R
IR-C-S
IR-St-L
IR-St-R
IR-St-S
UK-C-L
UK-C-R
UK-C-S
UK-St-L
UK-St-R
UK-St-S
B
PC 1 (42.89%)
PC 2 (24.57%)
C
Average NSAF Score (Sum of 100 proteins)
PC1 High
PC1 Low
Average NSAF Scores (Sum of 100 proteins)
PC2 High
PC2 Low
Iran-C-R
Iran-St-R
Iran-C-L
Iran-St-L
Iran-C-S
Iran-St-S
Cultivars
D
PC 1 (41.90%)
PC 2 (30.95%)
E
Average NSAF Scores (Sumof 100 proteins)
Averaged NSAF Scores (Sum of 100 proteins)
UK-C-R
UK-St-R
UK-C-L
UK-St-L
UK-C-S
UK-St-S

Figure S4

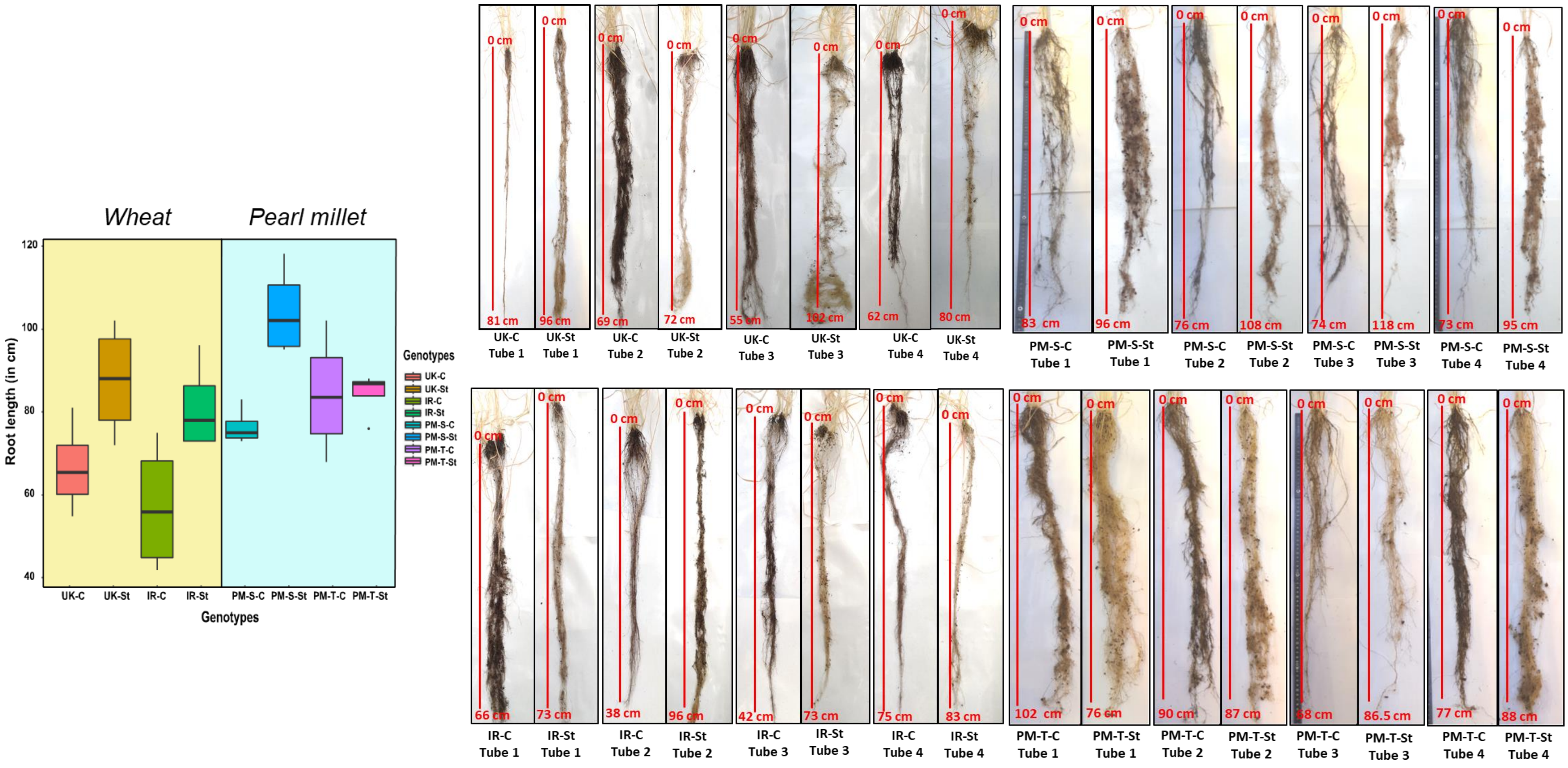
Wheat
Pearl millet
Root length (in cm)
Genotypes
UK-C
UK-St
IR-C
IR-St
PM-S-C
PM-S-St
PM-T-C
PM-T-St
UK-C Tube 1
UK-St Tube 1
UK-C Tube 2
UK-St Tube 2
UK-C Tube 3
UK-St Tube 3
UK-C Tube 4
UK-St Tube 4
PM-S-C Tube 1
PM-S-St Tube 1
PM-S-C Tube 2
PM-S-St Tube 2
PM-S-C Tube 3
PM-S-St Tube 3
PM-S-C Tube 4
PM-S-St Tube 4
IR-C Tube 1
IR-St Tube 1
IR-C Tube 2
IR-St Tube 2
IR-C Tube 3
IR-St Tube 3
IR-C Tube 4
IR-St Tube 4
PM-T-C Tube 1
PM-T-St Tube 1
PM-T-C Tube 2
PM-T-St Tube 2
PM-T-C Tube 3
PM-T-St Tube 3
PM-T-C Tube 4
PM-T-St Tube 4

Figure S5

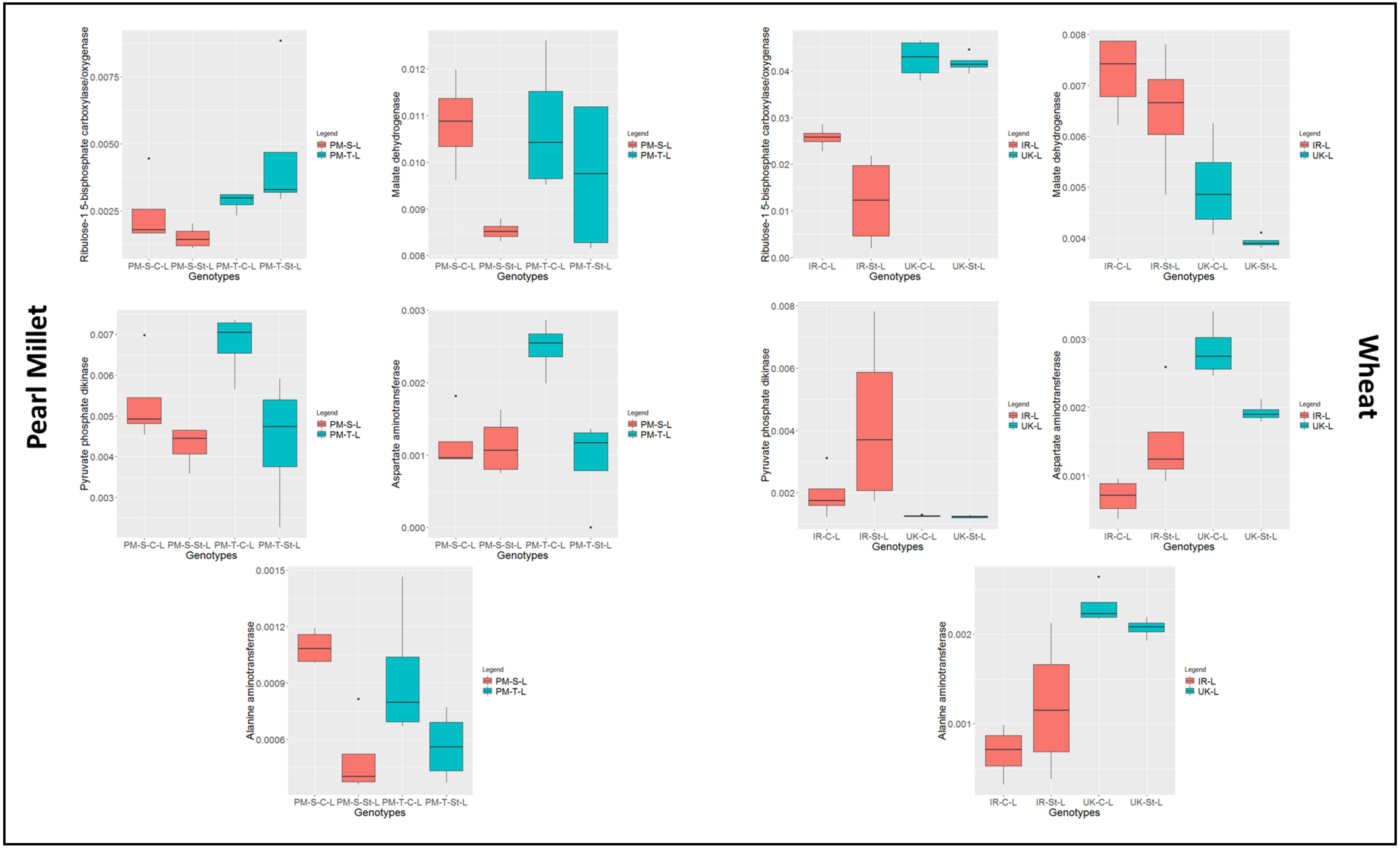
Pearl Millet
Wheat
Ribulose-1 5-bisphosphate carboxylase/oxygenase
Malate dehydrogenase
Pyruvate phosphate dikinase
Aspartate aminotransferase
Alanine aminotransferase
Genotypes
Legend
PM-S-L
PM-T-L
IR-L
UK-L
PM-S-C-L
PM-S-St-L
PM-T-C-L
PM-T-St-L
IR-C-L
IR-St-L
UK-C-L
UK-St-L

Figure S6

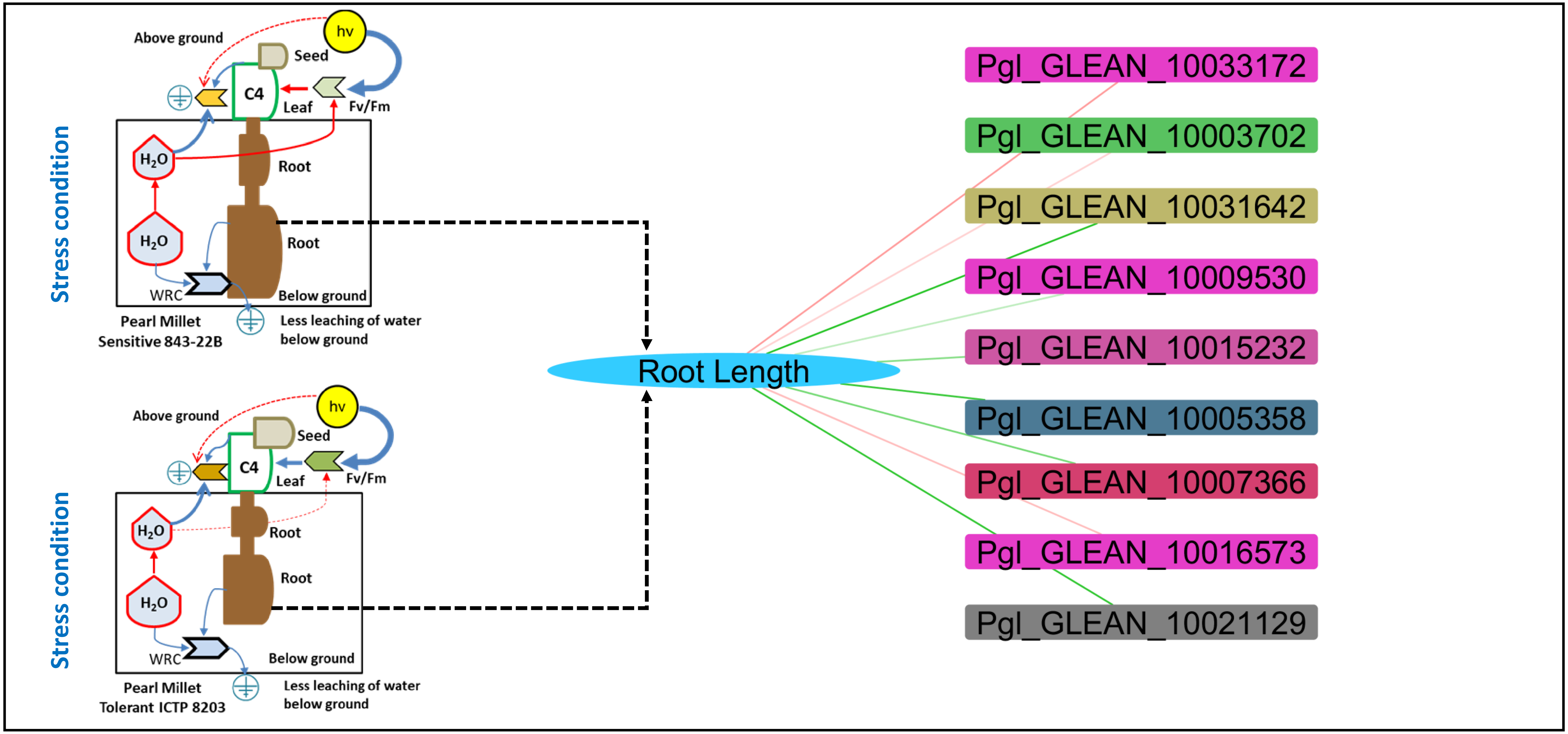
Above ground
hv
Seed
C4
Leaf
Fv/Fm
H2O
Root
H2O
Root
WRC
Below ground
Pearl Millet
Sensitive 843-22B
Less leaching of water
below ground
Stress condition
Above ground
hv
Seed
C4
Leaf
Fv/Fm
H2O
Root
H2O
Root
WRC
Below ground
Pearl Millet
Tolerant ICTP 8203
Less leaching of water
below ground
Stress condition
Root Length
Pgl_GLEAN_10033172
Pgl_GLEAN_10003702
Pgl_GLEAN_10031642
Pgl_GLEAN_10009530
Pgl_GLEAN_10015232
Pgl_GLEAN_10005358
Pgl_GLEAN_10007366
Pgl_GLEAN_10016573
Pgl_GLEAN_10021129

Figure S7

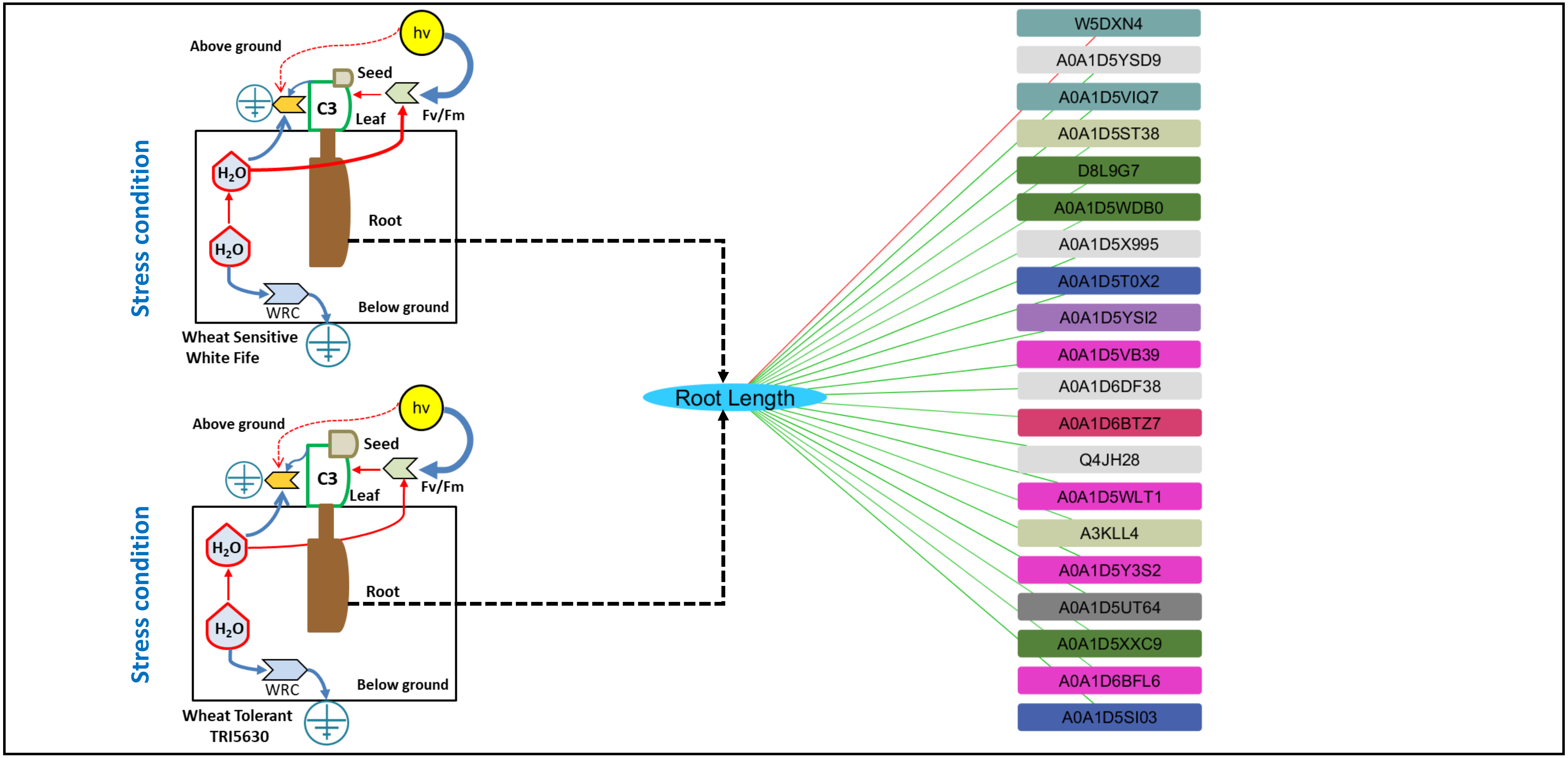
Stress condition
Above ground
hv
Seed
C3
Leaf
Fv/Fm
H_2O
H_2O
Root
WRC
Below ground
Wheat Sensitive
White Fife
Stress condition
Above ground
hv
Seed
C3
Leaf
Fv/Fm
H_2O
H_2O
Root
WRC
Below ground
Wheat Tolerant
TRI5630
Root Length
W5DXN4
A0A1D5YSD9
A0A1D5VIQ7
A0A1D5ST38
D8L9G7
A0A1D5WDB0
A0A1D5X995
A0A1D5T0X2
A0A1D5YSI2
A0A1D5VB39
A0A1D6DF38
A0A1D6BTZ7
Q4JH28
A0A1D5WLT1
A3KLL4
A0A1D5Y3S2
A0A1D5UT64
A0A1D5XXC9
A0A1D6BFL6
A0A1D5SI03

Figure S8

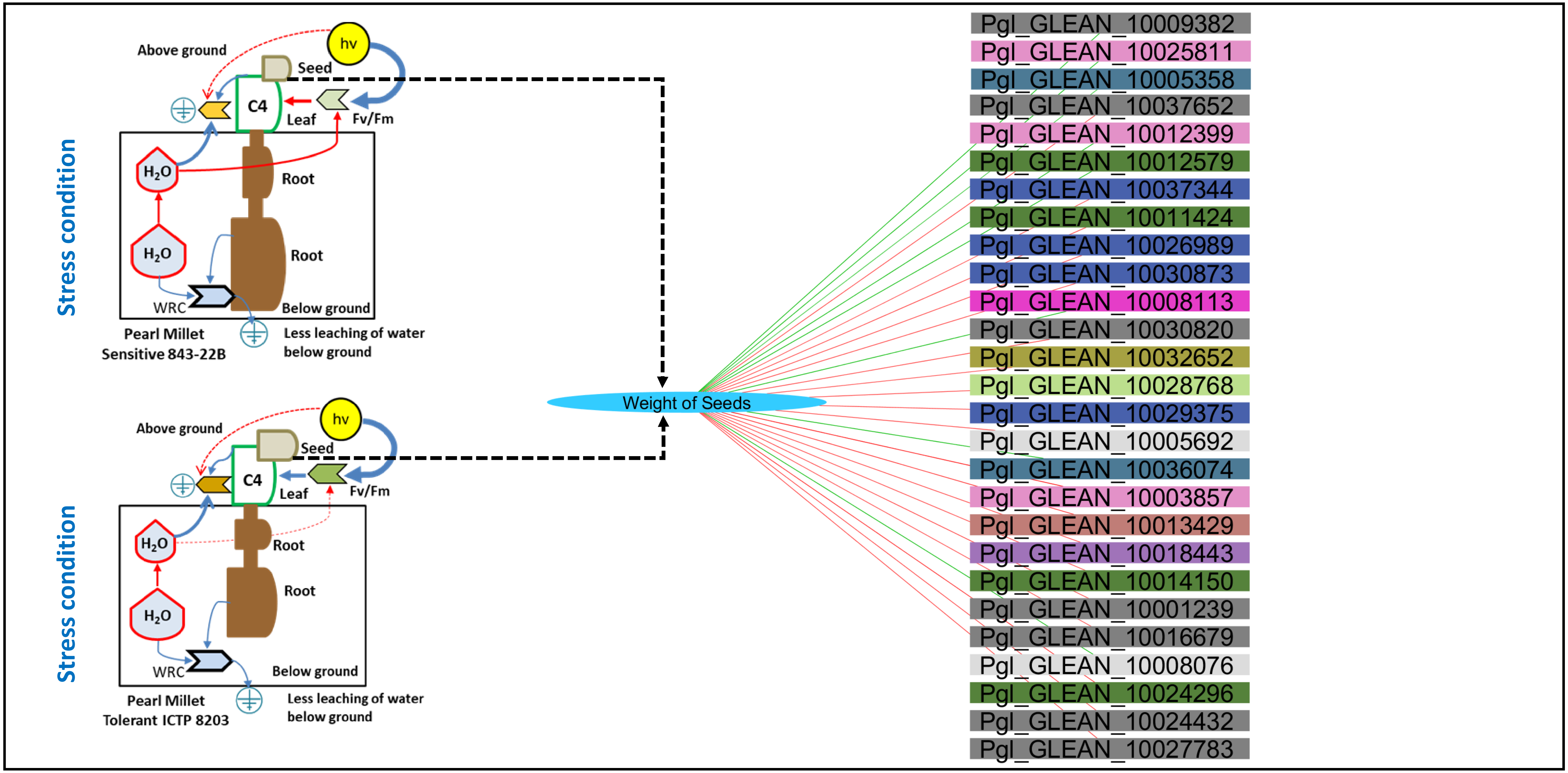

Stress condition
Above ground
hv
Seed
C4
Leaf
Fv/Fm
H_2O
Root
H_2O
Root
WRC
Below ground
Pearl Millet
Sensitive 843-22B
Less leaching of water
below ground
Stress condition
Above ground
hv
Seed
C4
Leaf
Fv/Fm
H_2O
Root
H_2O
Root
WRC
Below ground
Pearl Millet
Tolerant ICTP 8203
Less leaching of water
below ground
Weight of Seeds
Pgl_GLEAN_10009382
Pgl_GLEAN_10025811
Pgl_GLEAN_10005358
Pgl_GLEAN_10037652
Pgl_GLEAN_10012399
Pgl_GLEAN_10012579
Pgl_GLEAN_10037344
Pgl_GLEAN_10011424
Pgl_GLEAN_10026989
Pgl_GLEAN_10030873
Pgl_GLEAN_10008113
Pgl_GLEAN_10030820
Pgl_GLEAN_10032652
Pgl_GLEAN_10028768
Pgl_GLEAN_10029375
Pgl_GLEAN_10005692
Pgl_GLEAN_10036074
Pgl_GLEAN_10003857
Pgl_GLEAN_10013429
Pgl_GLEAN_10018443
Pgl_GLEAN_10014150
Pgl_GLEAN_10001239
Pgl_GLEAN_10016679
Pgl_GLEAN_10008076
Pgl_GLEAN_10024296
Pgl_GLEAN_10024432
Pgl_GLEAN_10027783

Figure S9

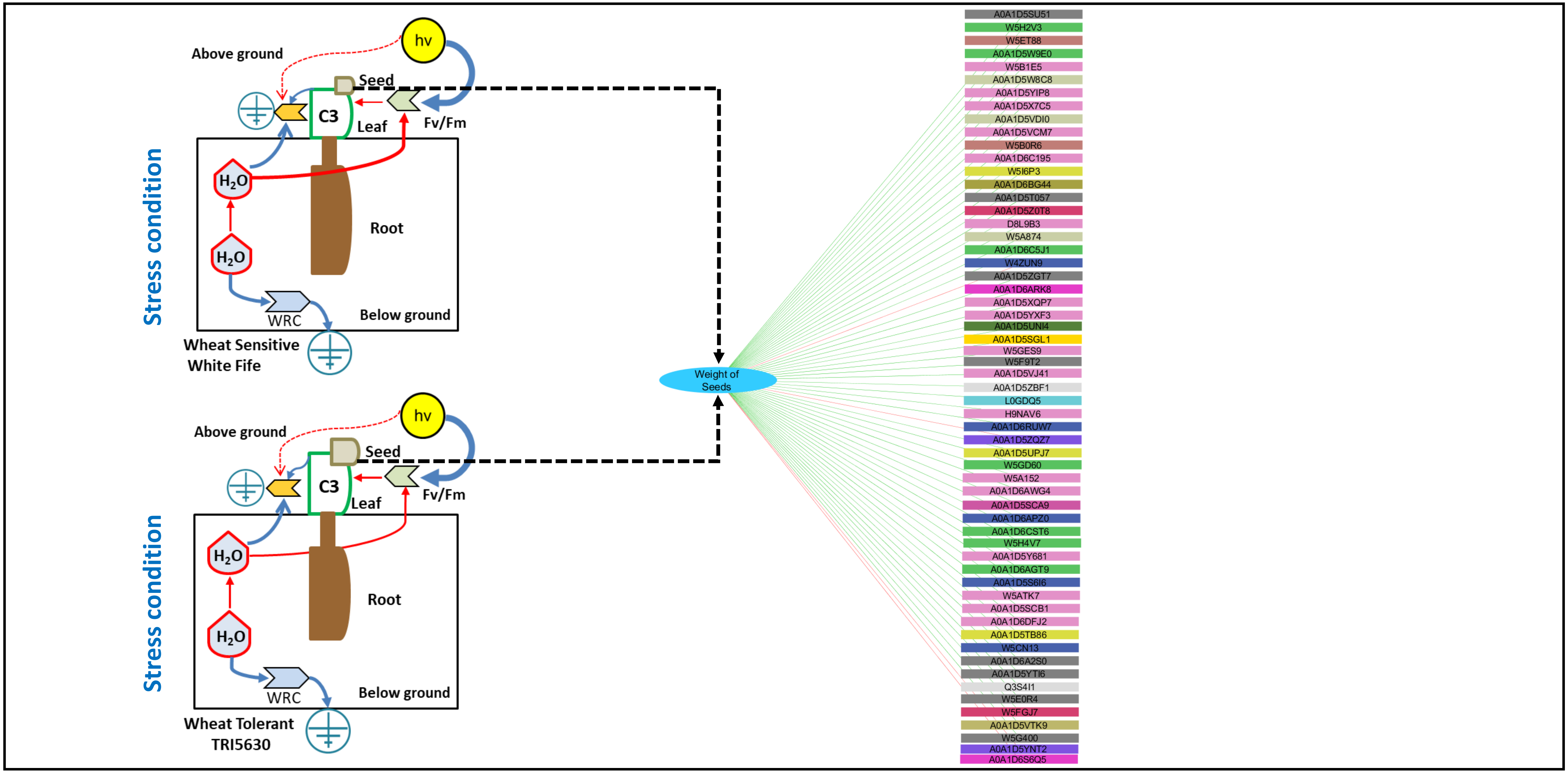
Stress condition
Above ground
hv
Seed
C3
Leaf
Fv/Fm
H_2O
H_2O
Root
WRC
Below ground
Wheat Sensitive
White Fife
Stress condition
Above ground
hv
Seed
C3
Leaf
Fv/Fm
H_2O
H_2O
Root
WRC
Below ground
Wheat Tolerant
TRI5630
Weight of Seeds
A0A1D5SU51
W5H2V3
W5ET88
A0A1D5W9E0
W5B1E5
A0A1D5W8C8
A0A1D5YIP8
A0A1D5X7C5
A0A1D5VDI0
A0A1D5VCM7
W5B0R6
A0A1D6C195
W5I6P3
A0A1D6BG44
A0A1D5T057
A0A1D5Z0T8
D8L9B3
W5A874
A0A1D6C5J1
W4ZUN9
A0A1D5ZGT7
A0A1D6ARK8
A0A1D5XQP7
A0A1D5YXF3
A0A1D5UNI4
A0A1D5SGL1
W5GES9
W5F9T2
A0A1D5VJ41
A0A1D5ZBF1
L0GDQ5
H9NAV6
A0A1D6RUW7
A0A1D5ZQZ7
A0A1D5UPJ7
W5GD60
W5A152
A0A1D6AWG4
A0A1D5SCA9
A0A1D6APZ0
A0A1D6CST6
W5H4V7
A0A1D5Y681
A0A1D6AGT9
A0A1D5S6I6
W5ATK7
A0A1D5SCB1
A0A1D6DFJ2
A0A1D5TB86
W5CN13
A0A1D6A2S0
A0A1D5YTI6
Q3S4I1
W5E0R4
W5FGJ7
A0A1D5VTK9
W5G400
A0A1D5YNT2
A0A1D6S6Q5

Figure S10
